# Combined high-quality metagenomics reveals off-target effects of albendazole, ivermectin-albendazole and moxidectin-albendazole on the human gut bacteria

**DOI:** 10.1038/s41522-026-01018-3

**Published:** 2026-05-29

**Authors:** Julian Dommann, Viviane P. Sprecher, Christian Beisel, Daniel Ballmer, Eveline Hürlimann, Jean T. Coulibaly, Jennifer Keiser, Pierre H. H. Schneeberger

**Affiliations:** 1 Swiss Tropical and Public Health Institute, Department of Medical Parasitology and Infection Biology, Allschwil, Switzerland; 2 University of Basel, Basel, Switzerland; 3 ETH Zürich, Department of Biosystems Science and Engineering, Basel, Switzerland; 4 Unité de Formation et de Recherche Biosciences, Université Félix Houphouët-Boigny, Abidjan, Côte d’Ivoire; 5 Centre Suisse de Recherches Scientifiques en Côte d’Ivoire, Abidjan, Côte d’Ivoire

**Keywords:** Diseases, Drug discovery, Microbiology

## Abstract

Human whipworm infections caused by *Trichuris trichiura* and *Trichuris incognita* remain a major public health problem, affecting over 400 million people globally and responding poorly to standard benzimidazole chemotherapy. Ivermectin-albendazole and moxidectin-albendazole have emerged as promising combination therapies, but recent in vitro evidence suggests that ivermectin and moxidectin may also affect gut bacteria. We therefore characterized their off-target effects on the gut microbiome in a randomized controlled trial including 204 *Trichuris* spp.-infected individuals in Côte d’Ivoire treated with albendazole (400 mg), ivermectin-albendazole (200 µg/kg/400 mg), or moxidectin-albendazole (8 mg/400 mg). By combining Illumina short reads and Nanopore long reads, we recovered over 800 high-quality metagenome-assembled genomes. Albendazole and moxidectin-albendazole induced taxonomic shifts with only mild functional consequences. In contrast, individuals receiving higher absolute ivermectin doses based on their bodyweight ( ≥ 15 mg) showed pronounced changes in taxonomic composition and microbial function, whereas the resistome remained largely stable. These findings confirm that ivermectin can exert antibacterial off-target effects in the human gut beyond those previously observed in vitro. Given its central role in parasite control, its broader microbiome effects warrant careful evaluation in future treatment strategies.

## Introduction

Soil-transmitted helminthiases, collectively classified as one of the 21 neglected tropical diseases, affect over 1.5 billion people worldwide^1,2^. Noteworthy, soil-transmitted helminthiases encompass the diseases caused by one or more species of gut-dwelling, parasitic nematodes. People living in resource-limited settings in the Global South are disproportionally affected. The soil-transmitted helminths (STHs) include the roundworm *Ascaris lumbricoides*, the threadworm *Strongyloides stercoralis*, the two whipworms *Trichuris trichiura* and *Trichuris incognita*^3,4^, and the two hookworms *Ancylostoma duodenale* and *Necator americanus*. Depending on the parasite species and severity of infection, symptoms differ. A heavy infection may include symptoms such as intestinal obstructions (*A. lumbricoides*), severe respiratory distress (*S. stercoralis*), rectal prolapse (*T. trichiura*) and severe anemia (hookworms)^5,6^. As all STHs require to spend a part of their development period in soil and transmission mainly relies on contact with egg- or larvae-infested soil, water or foods, long-term disease prevention focuses on improving hygiene and sanitation^7^. The short-term remedy is mass drug administration (MDA) of albendazole (ALB) and mebendazole (MBZ) without prior diagnosis. In the case of soil-transmitted helminthiases, MDA focuses primarily on children and may take place annually or biannually, depending on infection prevalence. Importantly, species-level parasite epidemiology is heterogeneous, and drug efficacies against the varying parasites differ^8,9^. For instance, single doses of ALB and MBZ are not sufficient to effectively reduce the burden and prevalence of *T. trichiura* infections^10^.

Like ALB and MBZ, ivermectin (IVM) is used in MDA programs to reduce the burden of lymphatic filariasis and onchocerciasis, two additional neglected tropical diseases^11,12^. IVM has recently been recommended as a partner-drug of ALB, potentially finding its way into STH MDA programs to broaden treatment efficacy against *T. trichiura*^13^. Moxidectin (MOX) is registered for onchocerciasis^14^ and might be used in combination with ALB for STH infections in the future^15–18^.

Structurally, both IVM and MOX, are macrocyclic lactones and therefore closely resemble the group of macrolide antibiotics. Owing to this fact, but also due to their shared habitat with the parasite in the host’s gastrointestinal tract, gut bacteria represent a tangible secondary target for IVM and MOX. In contrast to the parasites, the majority of gut bacteria make up an indispensable part of our gastrointestinal tract aiding in digestion, protection against pathogens and immune responses^19,20^. Disruption of the gut microbiome homeostasis could impede these critical functions. Given the harbored functional potential in the gut microbiome^21,22^ and the large-scale, continuous use of IVM - and at a later stage possibly MOX - in control programs, off-target activity may trigger bacterial drug resistance. While IVM resistance is well documented in veterinary helminths^23,24^, evidence from human parasites is more limited and often difficult to assess in MDA programmes in endemic, resource-limited settings. Yet reports consistent with reduced susceptibility or suboptimal response have been described, particularly for *Onchocerca volvulus*^25,26^. For human gut bacteria, clear evidence for IVM resistance is currently lacking, although this also may partly reflect the difficulty of studying bacterial resistance to these drugs in endemic, resource-limited MDA settings. Current evidence indicates wide-ranged antibacterial properties of the two anthelmintics and the phenotypical adaptation of bacteria to anthelmintics and multiple antibiotics after repeated anthelmintic exposure in vitro^27–29^. At the same time, analyses of the bacterial resistome after the trial “Macrolides Oraux pour Réduire les Décès avec un Oeil sur la Résistance I” (MORDOR I) demonstrated that biannual administration of the macrocyclic lactone azithromycin for two years to reduce childhood mortality increased the proportion of macrolide resistance genes in the gut microbiome^30,31^. Moreover, the proportion of macrolide resistance genes in the nasopharyngeal microbiome was elevated, demonstrating the role of the gut microbiome as a reservoir for antimicrobial resistance genes (ARGs) and emphasizing its key role in spreading ARGs^32^.

Considering wider use of IVM and MOX against STH and other helminth infections and the corresponding increase in administration, we aimed to identify the off-target effects of IVM and MOX on the bacterial gut microbiome characterized from stool samples collected in a randomized controlled phase 3 trial conducted in Côte d’Ivoire in 2021^33^. We generated high-quality metagenomes by combining short Illumina reads with long Nanopore reads for 204 participants infected with *Trichuris* spp. In the clinical study, these infections were diagnosed by microscopy as *T. trichiura*. Here, we use ‘*Trichuris* spp.’ in light of more recent evidence indicating that multiple *Trichuris* species, including *T. incognita*, may contribute to infections in Côte d’Ivoire^3,4^. To capture changes at both the participant level and across whole treatment arms, we combined a participant-level short-read workflow with a pooled treatment arm-level workflow. Participants were randomized in three treatment arms (ALB, IVM/ALB, and MOX/ALB), and stool examination took place at two timepoints (at baseline before treatment, BL; at follow-up after treatment, FU). In the subsequent analysis we compared, (i) species-level taxonomic composition, (ii) functional composition, as well as (iii) the resistome between BL and FU and across treatment arms.

## Results

### Taxonomic profiling reveals treatment-specific shifts of gut-communities for the ALB, IVM/ALB-low, IVM/ALB-high, and MOX/ALB arms

**Fig. 1:**
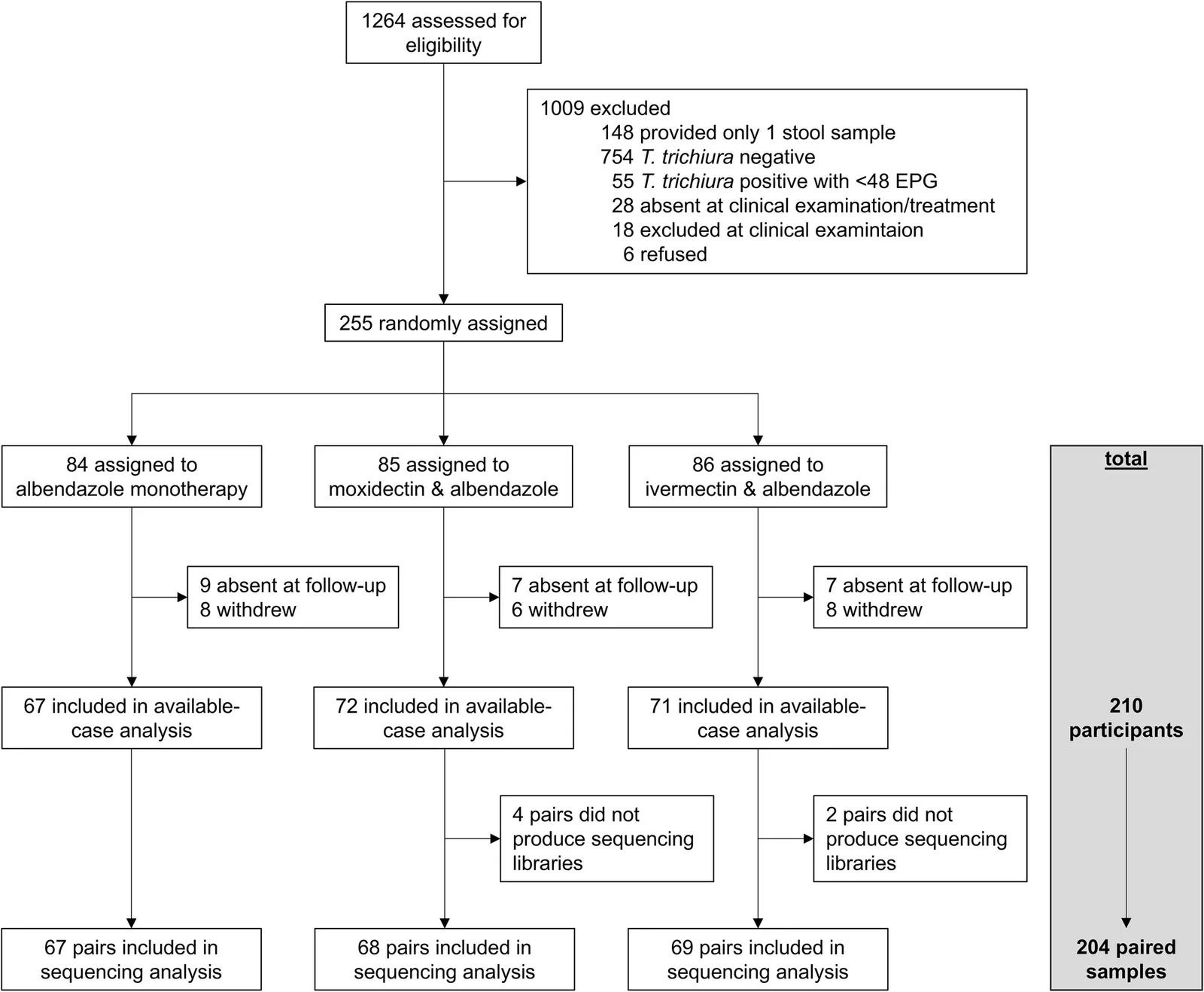
Trial profile. EPG eggs per gram of stool. The original trial profile is presented in Sprecher et al.^33^.

**Fig. 2:**
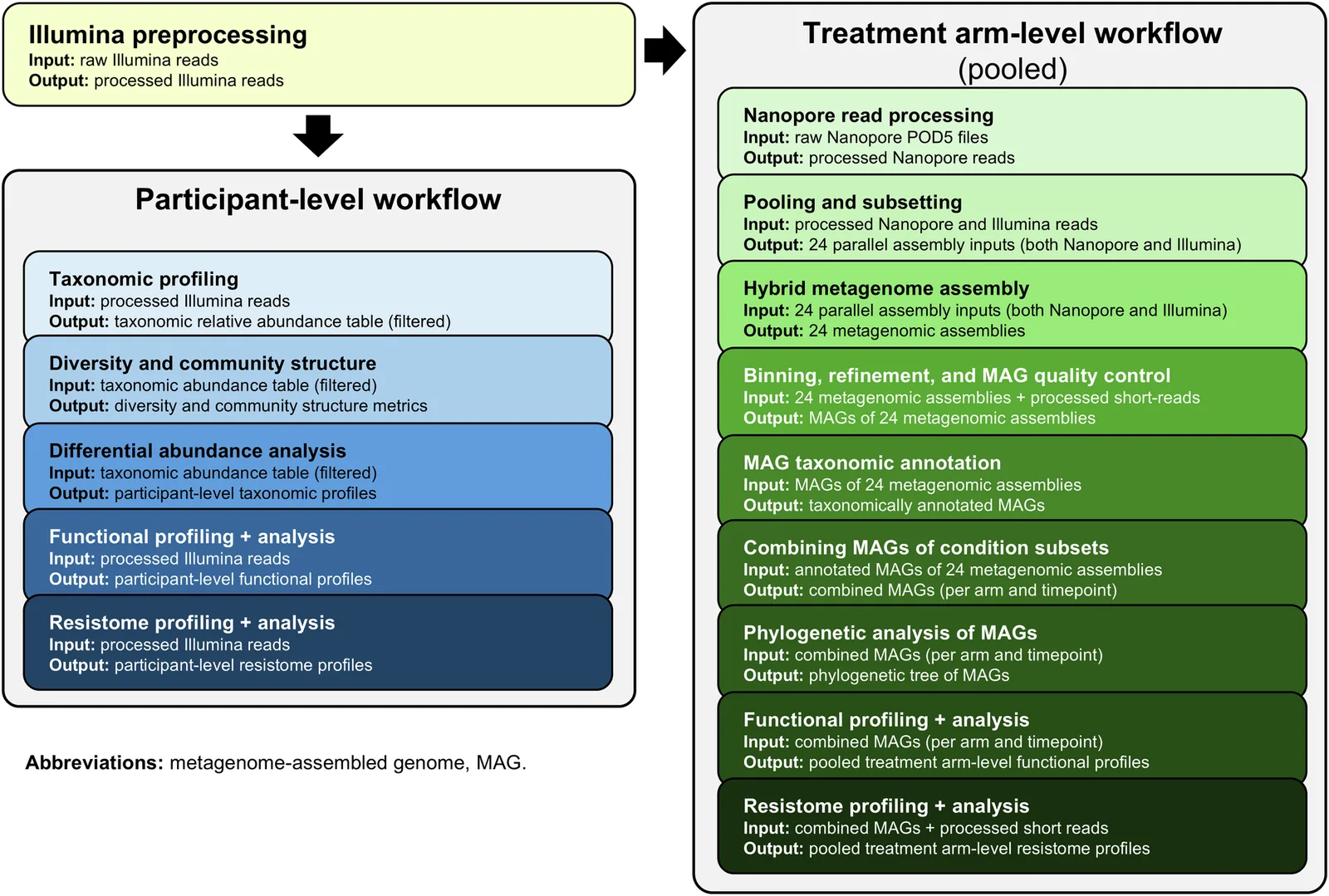
Abbreviated overview of the bioinformatics pipeline utilized to process both short reads and long reads yielding taxonomic, functional and resistome profiles on participant- and pooled treatmentarm-levels. A detailed overview of the bioinformatics pipeline is presented in Supplementary Fig. 1.

**Fig. 3:**
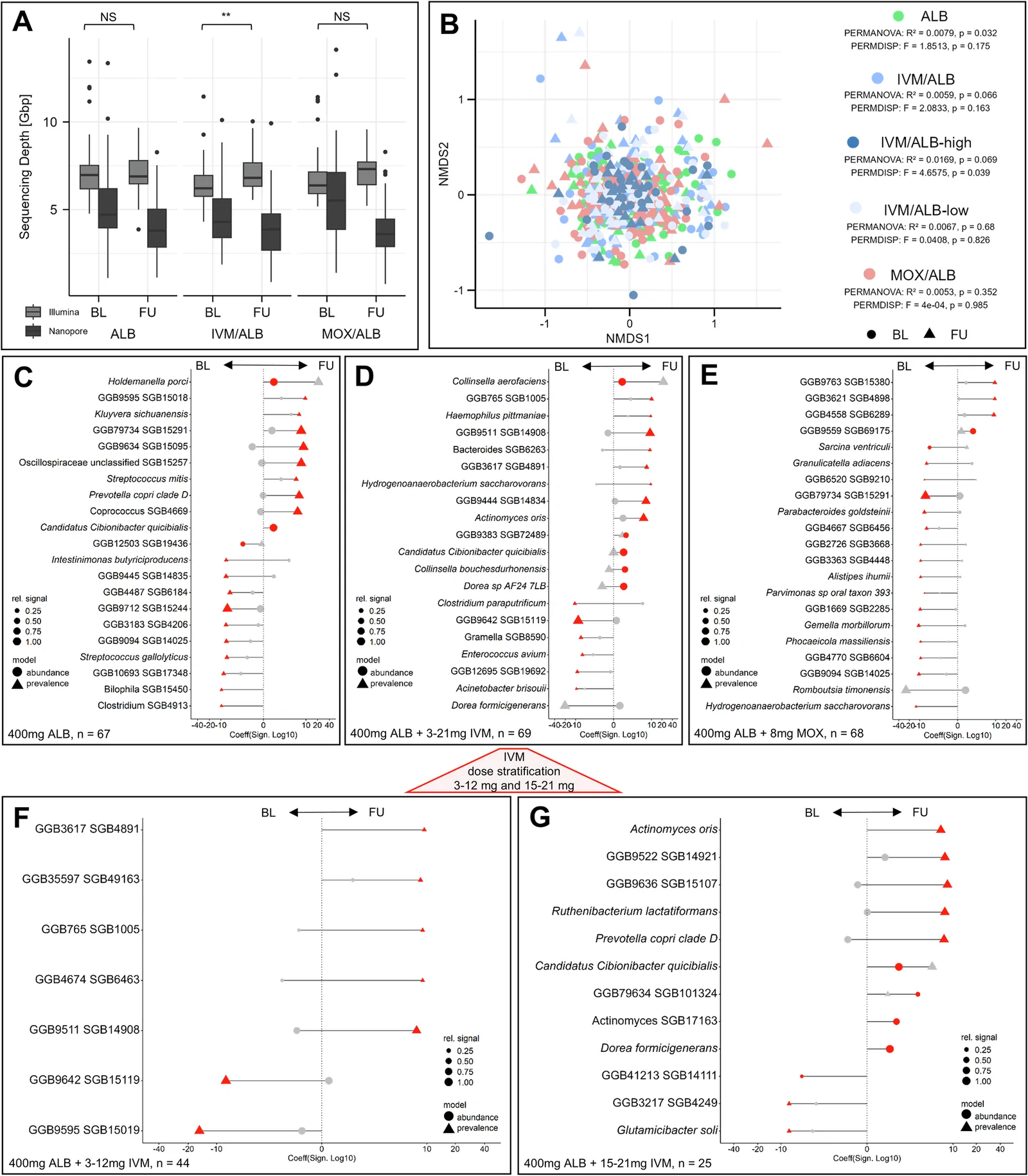
Taxonomic profiles in the albendazole (ALB), ivermectin-albendazole (IVM/ALB), and moxidectin-albendazole (MOX/ALB) arms at baseline (BL) and follow-up (FU). **A** Sequencing depths for 408 samples, 204 from BL and 204 from FU, sequenced on both the Illumina and Nanopore platforms. Boxplots show the median (center line), interquartile range (box), 1.5× IQR whiskers, and outliers (dots). For Illumina read depths, Bonferroni-corrected t-test *p*-values were 1 (ALB), 0.005 (IVM/ALB), and 0.192 (MOX/ALB). **B** Non-metric multidimensional scaling (NMDS) ordination based on Bray-Curtis dissimilarity of relative taxon abundances in paired BL and FU samples within each treatment arm. The IVM/ALB arm was further stratified by absolute administered IVM dose into IVM/ALB-low (3–12 mg) and IVM/ALB-high (15–21 mg). PERMANOVA: *p* = 0.032 (ALB), 0.066 (IVM/ALB), 0.069 (IVM/ALB-high), 0.680 (IVM/ALB-low), and 0.352 (MOX/ALB). PERMDISP: *p* = 0.175 (ALB), 0.163 (IVM/ALB), 0.039 (IVM/ALB-high), 0.826 (IVM/ALB-low), and 0.985 (MOX/ALB). **C**–**G** Species-level abundance and prevalence modelling comparing BL and FU within each treatment arm. Signals to the left of the x-axis centerline indicate association with BL, signals to the left of the x-axis centerline indicate association with FU. Only taxa reaching a “joint q-val” below 0.1 are shown. Data points with an “individual q-val” below 0.1 are shown in red. Abundance model coefficients (circles) are reported as log_2_(FU/BL), and prevalence model coefficients (triangles) as ln(OR). Relative signal was calculated as the number of samples in which the association was detected divided by the total number of samples for that arm and timepoint. **C** ALB; (**D**) IVM/ALB; (**E**) IVM/ALB-low (3–12 mg IVM); (**F**) MOX/ALB; (**G**) IVM/ALB-high (15–21 mg IVM).

We sequenced 67, 69, and 68 paired BL and FU samples from the ALB, IVM/ALB, and MOX/ALB arms, respectively (Fig. 1, Supplementary Table 1). Subsequent analyses were performed using two complementary bioinformatic workflows, summarized in Fig. 2 and described in detail in Supplementary Fig. 1. Paired-end Illumina sequencing yielded uniform read depths across all arms and timepoints (BL and FU), while Nanopore sequencing showed more variability (Fig. 3A). For subsequent analyses of the Nanopore data, 1 Gb per sample was used, excluding three samples that did not meet this threshold.

Diversity metrics based on Illumina data, including richness, Shannon diversity index (H′), and Berger-Parker dominance index (BP), showed no significant differences across treatment arms at BL (Supplementary Fig. 2, Supplementary Table 2). BL and FU were weakly to moderately correlated for richness and H′ across treatment arms (*ρ* = 0.28–0.55), whereas BP solely showed weak correlation in the IVM/ALB arm (*ρ* = 0.3; Supplementary Fig. 3). Phylum-level compositions remained stable between BL and FU within each treatment arm (Wilcoxon test: all *p* > 0.3; Supplementary Fig. 4). However, only the MOX/ALB arm showed a significant shift in fold-changes of core species between BL and FU (*p* = 0.024). In this arm, all core species were less prevalent at FU than at BL, except for *Anaerobutyricum soehngenii* (Supplementary Fig. 5). No significant core species-level changes between BL and FU were observed in the ALB (*p* = 0.779) or IVM/ALB (*p* = 0.365) arms.

Because IVM regimen is weight-based (200 µg/kg bodyweight), we stratified the IVM/ALB arm by absolute dose: IVM/ALB-low (3–12 mg, *n* = 44) and IVM/ALB-high (15–21 mg, *n* = 25), based on the number of 3 mg tablets administered. Diversity metrics remained unchanged between these subgroups at BL (Supplementary Fig. 6, Supplementary Table 2). For richness and H′, BL and FU were moderately to strongly correlated in the IVM/ALB-low arm (*ρ* = 0.54–0.65), but not in the IVM/ALB-high arm. BP showed no correlation in either IVM/ALB arm (Supplementary Fig. 7). A PERMANOVA based on Bray-Curtis dissimilarity indicated modest differences in community composition between BL and FU up in the ALB arm (R² = 0.0079, *p* = 0.032), the overall IVM/ALB arm (R² = 0.0059, *p* = 0.066) and the IVM/ALB-high arm (R² = 0.0169, *p* = 0.069) (Fig. 3B). PERMDISP analysis indicated significant dispersion only in the IVM/ALB-high group (F = 4.658, *p* = 0.039), suggesting that the differences observed in the ALB arm and the overall IVM/ALB arm reflect shifts in community composition, whereas in the IVM/ALB-high arm larger differences between BL and FU were seen only in some participants.

Species-level comparisons of BL and FU within each treatment arm, based on MetaPhlAn-derived taxonomic profiles, showed the clearest changes in the IVM/ALB-high arm (Fig. 3G). At FU, *Dorea formicigenerans* was more abundant than at BL (log₂[FU/BL] = 0.9, *q* = 0.05), while *Actinomyces oris* and *Ruthenibacterium lactatiformans* were more prevalent (ln[OR] = 6.8, *q* = 0.10; ln[OR] = 7.7, *q* = 0.05). In contrast, the IVM/ALB-low arm (Fig. 3E) showed higher prevalence at FU than at BL for one taxon, SGB14908, whose closest assigned relative fell within the Clostridiaceae (ln[OR] = 7.7, *q* < 0.001). The MOX/ALB arm (Fig. 3F) showed higher abundance at FU of SGB69175, for which the closest relative was unknown (log₂[FU/BL] = 1.6, *q* = 0.08), and lower prevalence at FU than at BL of SGB15291, whose closest assigned relative fell within the Ruminococcaceae (ln[OR] = −6.7, *q* < 0.001). ALB treatment (Fig. 3C) was associated with higher abundance of *Holdemanella porci* at FU (log₂[FU/BL] = 0.8, *q* = 0.03), as well as higher prevalence of *Streptococcus mitis* and *Prevotella copri* (ln[OR] = 5.4, *q* = 0.06; ln[OR] = 6.5, *q* < 0.001). Random Forest analysis supported these findings, identifying *H. porci*, *Collinsella aerofaciens*, *D. formicigenerans*, and SGB69175 as key discriminants between BL and FU within treatment arms (Supplementary Figs. 8–12). Collectively, ALB and IVM/ALB-high treatment were associated with the clearest species-level differences between BL and FU, underscoring their specific yet broad impacts at the species level.

### Functional profiling of treatment-stratified, pooled metagenomes reveals profound differences between timepoints for the ALB and IVM/ALB-high arms

**Fig. 4:**
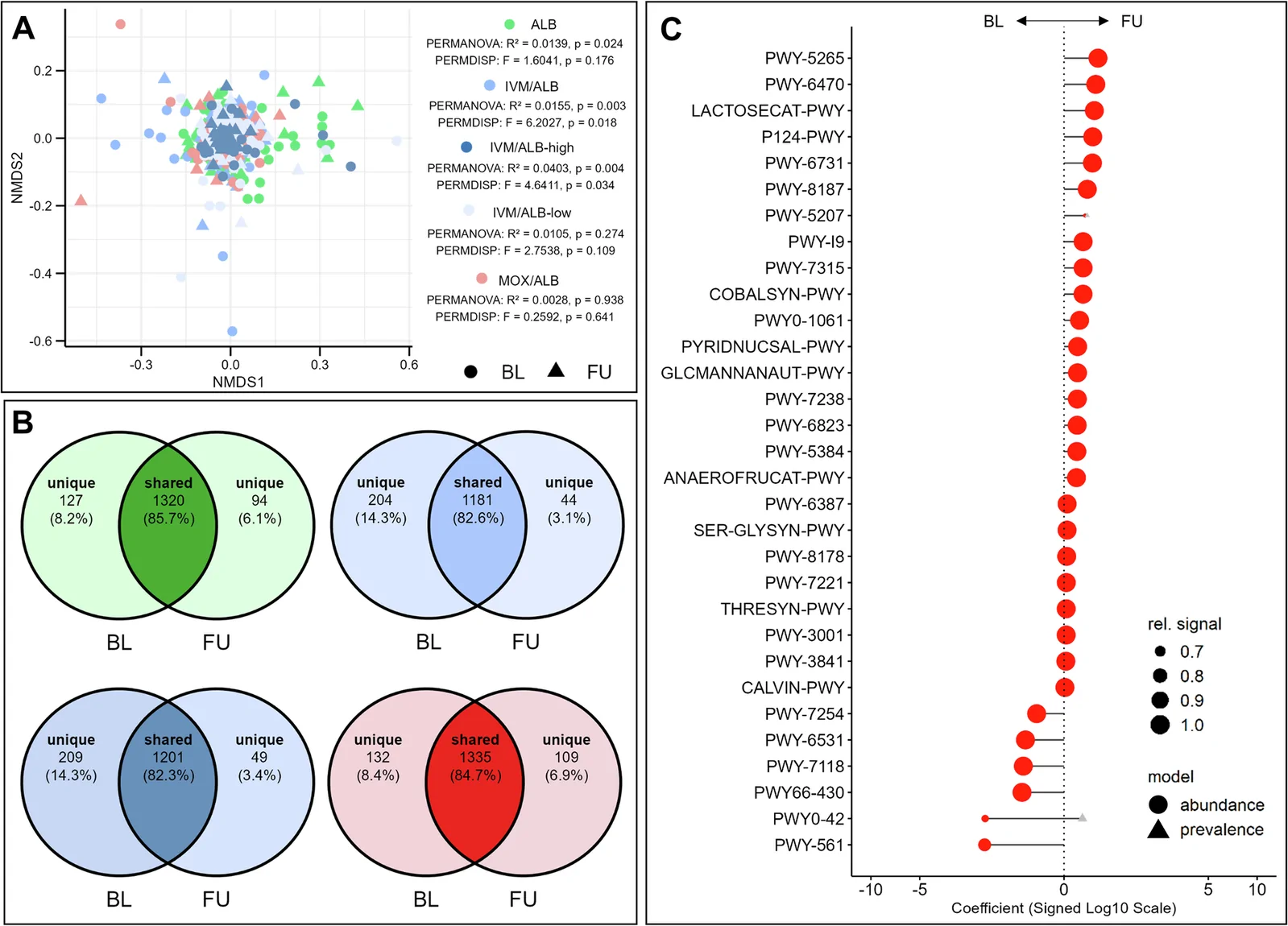
Functional profiles in the albendazole (ALB), ivermectin-albendazole (IVM/ALB), and moxidectin-albendazole (MOX/ALB) arms at baseline (BL) and follow-up (FU). **A** Non-metric multidimensional scaling (NMDS) ordination based on Bray-Curtis dissimilarity of relative pathway abundances in BL and FU samples within each treatment arm. The IVM/ALB arm was further stratified by absolute administered IVM dose into IVM/ALB-low (3–12 mg IVM) and IVM/ALB-high (15–21 mg IVM). PERMANOVA: *p* = 0.024 (ALB), 0.003 (IVM/ALB), 0.004 (IVM/ALB-high), 0.274 (IVM/ALB-low), and 0.938 (MOX/ALB). PERMDISP: *p* = 0.176 (ALB), 0.018 (IVM/ALB), 0.034 (IVM/ALB-high), 0.109 (IVM/ALB-low), and 0.641 (MOX/ALB). **B** Absolute and relative numbers of shared and unique enzyme commission (EC) numbers in metagenome-assembled genomes (MAGs) between BL and FU within each treatment arm. **Top**: ALB (**left**), IVM/ALB-low (**right**). **Bottom**: IVM/ALB-high (**left**), MOX/ALB (**right**). **Top:** ALB (left), IVM/ALB-low (right). **Bottom:** IVM/ALB-high (left), MOX/ALB (right). **C** Pathway abundance and prevalence modelling comparing BL and FU within the IVM/ALB-high arm after filtering to retain only associations with the 20% lowest and 20% highest coefficients and a relative signal > 0.5. Signals to the left of the x-axis centerline indicate enrichment with BL, signals to the left of the x-axis centerline indicate enrichment with FU. Relative signal was calculated as the ratio of samples in which the association was detected to the total number of samples for that arm and timepoint. Only pathways with a “joint q val” < 0.1 are shown. Pathways with an “individual q val” < 0.1 are shown in red. Abundance model coefficients are reported as log_2_(FU/BL), and prevalence model coefficients as ln(OR).

To link taxonomic shifts to potential biological relevance, we next compared functional profiles between BL and FU within each treatment arm. Bray-Curtis dissimilarity of pathway abundances (Fig. 4A) showed a small but significant difference between BL and FU in the ALB arm (R² = 0.0139, *p* = 0.024), no significant difference in the IVM/ALB-low (R² = 0.0105, *p* = 0.274) or MOX/ALB arms (R² = 0.0028, *p* = 0.938), and the strongest difference in the IVM/ALB-high arm (R² = 0.0403, *p* = 0.004). PERMDISP suggested that the difference in the ALB arm reflected a more consistent shift across participants (F = 1.604, *p* = 0.176), whereas the difference in the IVM/ALB-high arm was accompanied by significant dispersion (F = 4.641, *p* = 0.034), indicating stronger participant-level variation. MetaCyc reaction-level beta-diversity (Supplementary Fig. 13) showed the same overall pattern, with significant differences between BL and FU in the ALB (R² = 0.0117, *p* = 0.035) and IVM/ALB-high arms (R² = 0.0374, *p* = 0.003), but not in the IVM/ALB-low (R² = 0.0127, *p* = 0.146) or MOX/ALB arms (R² = 0.0049, *p* = 0.573). PERMDISP analysis again indicated significant dispersion only in the IVM/ALB-high arm (F = 4.123, *p* = 0.048), supporting greater participant-level variation in this group. Together, these results indicate that functional potential differed most clearly between BL and FU in the IVM/ALB-high arm, with smaller but significant differences also observed in the ALB arm at both the pathway and reaction levels.

To contextualize functional differences between BL and FU at the genome level, we analyzed MAGs within each treatment arm. At BL, binning yielded 135, 100, 91, and 130 high-quality MAGs (completeness > 95%, contamination < 5%) in the ALB, IVM/ALB-low, IVM/ALB-high, and MOX/ALB arms, respectively. At FU, we recovered 116, 103, 80, and 121 MAGs in the same arms. At each timepoint, a core set of MAGs was shared across treatment arms (18% at BL; 16% at FU), alongside smaller sets unique to individual arms (9–15% at BL; 6-13% at FU; Supplementary Fig. 14. Comparisons of BL and FU within each treatment arm showed that at least 40% of MAGs were shared across timepoints, consistent with a stable core microbiome (Supplementary Fig. 15), although each arm also contained timepoint-specific MAGs ( ≥ 20%). Treatment-specific phylogenetic trees integrating MAGs from BL and FU (Supplementary Figs. 16–19) likewise showed broad phylogenetic diversity and several clades shared between timepoints in all treatment arms.

Gene content similarity, assessed by Enzyme Commission (EC) annotation (Fig. 4B), also showed high overlap in enzymatic functions between BL and FU within each treatment arm (82–86%), indicating that core microbiome functions were largely preserved after treatment. Clearer functional differences between BL and FU emerged from comparisons of pathway abundance and prevalence within each treatment arm (Fig. 4C, Supplementary Fig. 20). After filtering for pathways within the top and bottom 20% of model coefficients (Supplementary Fig. 21), the ALB arm showed four pathways with higher abundance or prevalence at FU than at BL: CDP-diacylglycerol biosynthesis I (PWY-5667), CDP-diacylglycerol biosynthesis II (PWY0-1319), starch degradation III (PWY-6731), and 1,5-anhydrofructose degradation (PWY-6992), all related to phospholipid or carbohydrate metabolism. The IVM/ALB-high arm showed the largest and most diverse set of pathways with higher abundance or prevalence at FU, spanning core metabolism (e.g., LACTOSECAT-PWY, PWY-7238, PWY-5384), nucleotide turnover (e.g., PWY-7221), amino acid metabolism (e.g., PWY-I9, PWY-8187), cofactor biosynthesis (e.g., PWY-6823, PWY-5207), and multiple peptidoglycan biosynthesis pathways (e.g., PWY-5265, PWY-6387, PWY-6470). In the MOX/ALB arm, three pathways were more abundant or prevalent at FU than at BL, including two related to secondary metabolite biosynthesis (PWY-992, PWY-5910). No significantly enriched pathways were detected in the IVM/ALB-low arm.

To further resolve these differences between BL and FU, we examined MetaCyc reaction-level changes (Supplementary Fig. 22), filtered to the top and bottom 5% of model coefficients (Supplementary Fig. 23). In the ALB arm, more than 20 differentially abundant reactions were associated with enzymes annotated with the monooxygenase activity EC 1.14.14.1. The IVM/ALB-high arm again showed broader diversity of differences, including a stress-activated kinase (2.7.11.24-RXN), two hydrolases (3.1.3.77-RXN and 3.2.1.156-RXN), and reactions involved in lactose- or maltose-specific phosphotransferase systems (RXN-15158, RXN-15159, RXN-15166, and RXN-15167), among others. By contrast, the IVM/ALB-low arm showed differential prevalence of only a small set of reactions concerning succinylase and inulinase activity (SUCCGLUDESUCC-RXN and EC 3.2.1.7), whereas the MOX/ALB arm was associated with a lyase (PROTOCATECHUATE-DECARBOXYLASE-RXN) and two transferases (GSDEADENYLATION-RXN and RXN-12464). Taken together, these findings reveal nuanced, treatment-specific metabolic responses of the gut microbiome to these anthelmintic interventions, with IVM/ALB-high inducing particularly strong and systemic functional reprogramming, likely driven by microbial restructuring or altered host–microbiota interactions.

### Resistome analysis of arm-specific metagenomic communities reveals limited differences between study timepoints

**Fig. 5:**
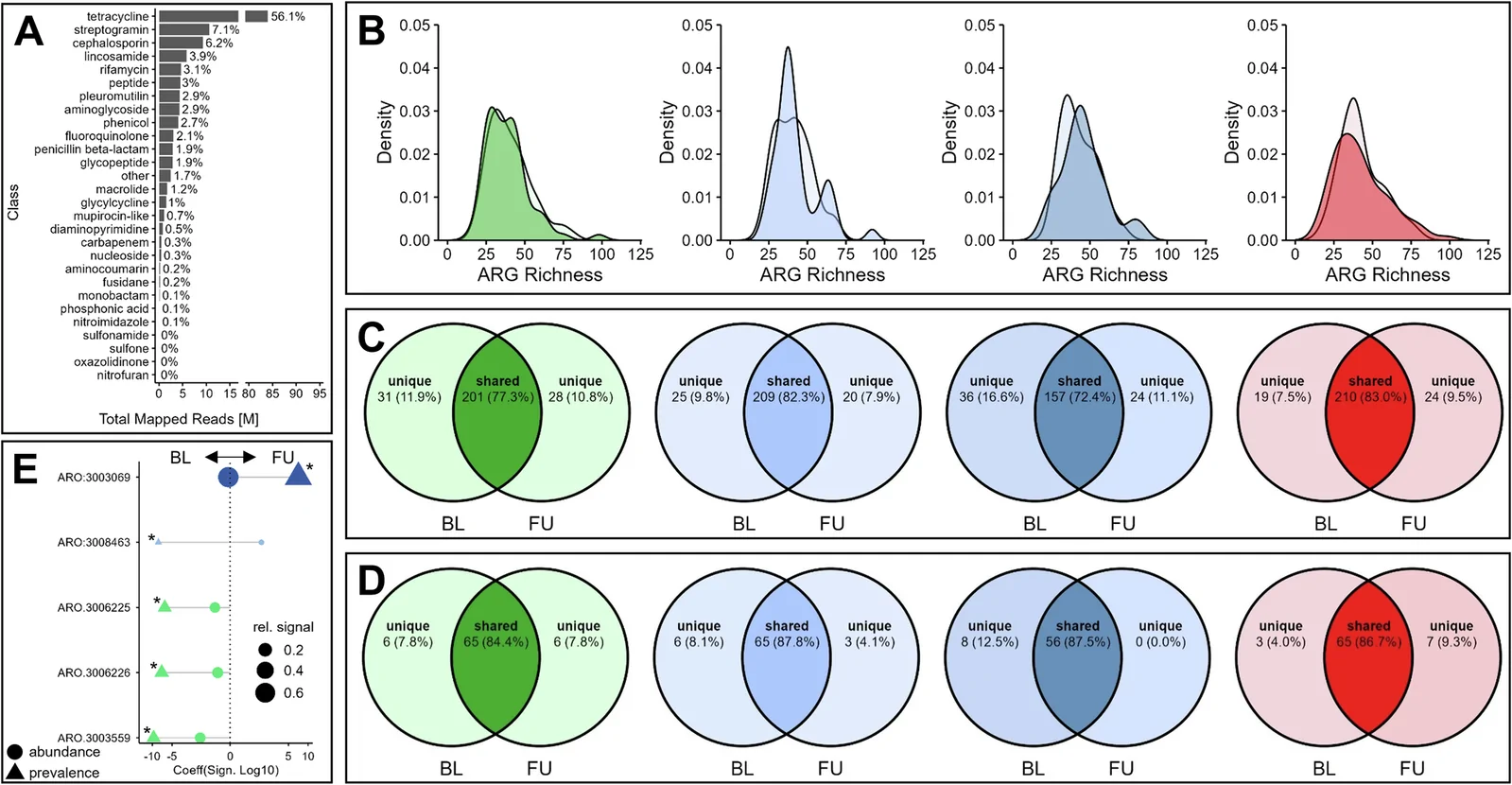
Resistome profiles based on Illumina sequencing reads for albendazole (ALB), ivermectin-albendazole (IVM/ALB) and moxidectin-albendazole (MOX/ALB) cohorts at baseline (BL) and follow-up (FU). **A** Absolute abundance of antimicrobial resistance genes (ARGs) per antibiotic class in the whole study cohort at BL. **B** ARG richness for BL and FU cohorts represented in a density function. **Left to right:** ALB, IVM/ALB-low, IVM/ALB-high, MOX/ALB. Darker colors represent the FU content, lighter colors represent the BL content. **C**, **D** Absolute and relative number of shared and unique ARGs (**C**) and ARG classes (**D**) between BL and FU timepoints. Left to right: ALB IVM/ALB-low, IVM/ALB-high, MOX/ALB. **E** ARG abundance and prevalence modelling comparing BL and FU within each treatment arm. Signals to the left of the x-axis centerline indicate association with BL, signals to the left of the x-axis centerline indicate association with FU. Only ARGs with a “joint q val” < 0.1 are shown. Data points with an “individual q val” < 0.1 are marked with an asterisk. Abundance model coefficients are reported as log_2_(FU/BL), and prevalence model coefficients as ln(OR). Relative signal was calculated as the ratio of samples in which the association was detected to the total number of samples for that arm and timepoint. Green, ALB light blue, IVM/ALB-low; dark blue, IVM/ALB-high.

At baseline, the gut resistome of the overall cohort was dominated by ARGs targeting tetracyclines (56.1%), followed by streptogramins (7.1%), cephalosporins (6.2%), lincosamides (3.9%), and rifamycins (3.1%) while macrolide-related ARGs which are of interest because of the structural similarity between macrolides antibiotics and IVM and MOX accounted for 1.2% of the total ARG pool (Fig. 5A). ARG richness increased slightly from BL to FU across treatment arms, although only a few ARGs exceeded a richness threshold of > 75 (Fig. 5B). Within each treatment arm, most ARGs were shared between BL and FU (ALB: 77.3%, IVM/ALB-low: 82.3%, IVM/ALB-high: 72.4%, MOX/ALB: 83.0%), with smaller fractions unique to BL (7.5–16.6%) or FU (7.9–11.1%) (Fig. 5C). ARG beta-diversity remained stable between BL and FU within each treatment arm (Supplementary Fig. 24), and most ARG classes were likewise conserved across timepoints (Fig. 5D; shared: 84.4–87.8%, BL-unique: 7.8–12.5%, FU-unique: 0.0–9.3%). Comparisons of ARG prevalence between BL and FU within each treatment arm (Fig. 5E) identified only a few significant differences. In the IVM/ALB-low arm, the beta-lactamase *OXA-1044* was approximately 3300-fold less prevalent at FU than at BL (ln[OR] = −8.1, *q* = 0.001). In the ALB arm, *CepA-44, CepA-49*, and *cepA* were more prevalent at BL than at FU, corresponding to more than 600-fold lower prevalence at FU (all ln[OR] < −6.4, *q* < 0.001). In the IVM/ALB-high arm, *vanXY-G* was approximately 1300-fold more prevalent at FU than at BL (ln[OR] = 7.2, *q* = 0.02). Notably, the macrolide efflux pump *macB* also showed markedly higher prevalence at FU in both the IVM/ALB-high and MOX/ALB arms, although these changes were not significant (ln[OR] = 7.4, *q* = 0.49 and ln[OR] = 11.8, *q* = 0.24, respectively).

**Fig. 6:**
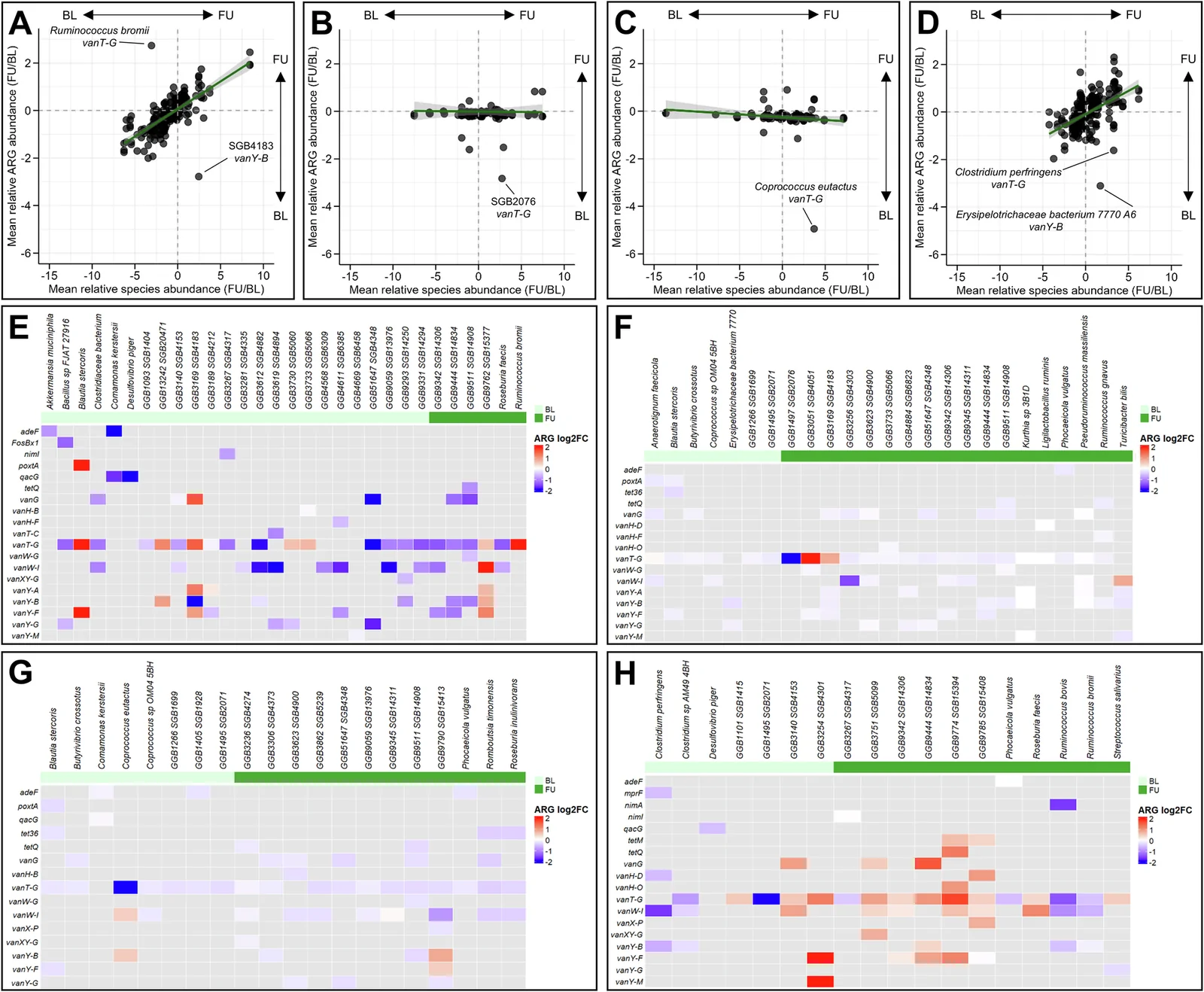
Resistome profiles of metagenomic assembled genomes for albendazole (ALB), ivermectin-albendazole (IVM/ALB) and moxidectin-albendazole (MOX/ALB) cohorts at baseline (BL) and follow-up (FU). **A**–**D** Scatter of mean log_2_-transformed antimicrobial resistance gene (ARG) abundance fold-change originating from metagenome-assembled genomes (MAGs) and the corresponding mean log_2_-transformed species abundance fold-change from MetaPhlAn profiles (Fig. 3) between the BL and FU timepoints (FU/BL). The corresponding species abundances were matched with the ARG abundances by applying the MetaPhlAn taxonomy to the MAGs. **A** ALB (**B**) IVM/ALB-low (**C**). IVM/ALB-high (**D**). MOX/ALB. **E**–**H** Mean log_2_-transformed ARG abundance fold-changes (FU/BL) for the MAGs exerting a mean log_2_-transformed species abundance fold-change (FU/BL) > 2 or < −2. We used the existing MetaPhlAn profiles (Fig. 3) to calculate mean abundance fold-changes (FU/BL) for each species and subsequently applied the MetaPhlAn taxonomy to the MAGs. (**E**) ALB (**F**) IVM/ALB-low (**G**) IVM/ALB-high (**H**). MOX/ALB.

To assign changes in the pooled treatmentarm-level resistome to specific microbial host MAGs, we next examined ARG changes within high-quality MAGs ( > 95% completeness, < 5% contamination) and compared mean ARG abundance between BL and FU with the abundance changes of the corresponding host taxa (Fig. 6A–D). Across treatment arms, ARG abundance largely mirrored host MAG abundance, suggesting limited gene-specific selection, with only a few exceptions (residual > 2). In the ALB arm (Fig. 6A), *vanT-G* in *Ruminococcus bromii* and *vanY-B* in SGB4183, whose closest genomic relative is *Ruminococcus* sp., deviated from this overall pattern. In both the IVM/ALB-low and IVM/ALBhigh arms (Figs. 6B and 5C), *vanT-G* also diverged from host MAG abundance changes. This occurred in SGB2076, whose closest relative is *Prevotella* sp., and in *Coprococcus eutactus*, respectively. In both cases, *vanT-G* was disproportionately enriched at BL relative to the corresponding host MAGs. In the MOX/ALB arm, *vanT-G* and *vanY-B* were less abundant than expected based on the abundance of their parent MAGs, *Clostridium perfringens* and *Erysipelotrichaceae* bacterium 7770 A6, respectively. In the ALB arm, taxa with strong abundance differences between BL and FU ( > 4-fold, |log_2_FC | > 2) included *Akkermansia muciniphila* carrying *adeF*, *Comamonas kerstersii* carrying *adeF* and *qacG*, and *Desulfovibrio piger* carrying *qacG*. All were more abundant at BL. Although *Blautia stercoris* was also more abundant at BL, its associated *poxtA* gene was more abundant at FU. In both the IVM/ALB-low and IVM/ALB-high arms, no additional strong ARG abundance differences were detected among MAGs enriched at either timepoint (Fig. 6F, G). In the MOX/ALB arm, *Clostridium perfringens* and *Desulfovibrio piger* were more abundant at BL, together with *mprF* and vancomycin resistance genes. At FU, vancomycin resistance genes varied in abundance and were associated with higher levels of *Phocaeicola vulgatus*, *Roseburia faecis*, *Ruminococcus* spp., and *Streptococcus salivarius*. Overall, although a small number of ARGs showed timepoint-specific deviations in selected taxa, the overall gut resistome remained largely stable between BL and FU across all treatment arms.

## Discussion

Given the limited arsenal of anthelmintic treatment options, combination therapies involving the macrocyclic lactones IVM and MOX offer a well needed perspective for a better treatment option for STH infections. Beyond their well-established roles in treating parasitic nematode infections, IVM in particular is investigated for malaria transmission control through repeated high-dose regimens (up to 600 µg/kg)^34,35^. However, despite this promising potential, current evidence raises several concerns. First, the efficacy of macrocyclic lactone-benzimidazole combination treatments against STH infections exhibits notable geographical and species-specific variability^33,36–38^. Moreover, recent in vitro studies have highlighted the broad-spectrum antibacterial properties of these compounds^27,28^. Yet comprehensive data on their potential off-target effects in human populations remain scarce.

For IVM, previous investigations on the gut microbiome have been constrained in terms of sample size, taxonomic resolution (genus-level), the use of less widely used partner drugs, such as tribendimidine, and a direct comparison to the current standard of care (e.g., ALB)^39,40^. In this study, we address these gaps by providing the first in-depth characterization of off-target taxonomic and functional changes following ALB, IVM/ALB or MOX/ALB treatment in a large cohort of *Trichuris* spp.-infected individuals in Côte d’Ivoire. We combined a participant-level short-read workflow for taxonomic, functional, and resistome profiling with a pooled, treatment arm-level approach to generate high-quality MAGs providing additional context for ARG-related insights. Indeed, this allowed us to link ARGs to specific microbial taxa across more than 800 high-quality MAGs and to provide both a reference and resource for future microbiome and AMR studies, especially in helminth-endemic settings.

A general challenge in interpreting microbiome changes in this study is that differences between BL and FU may reflect both direct effects of treatment on gut bacteria and indirect effects mediated by parasite clearance or changes in other members of the gut microbiome. This is particularly relevant in our setting because *A. lumbricoides*, another STH, is endemic in Côte d’Ivoire and is highly susceptible to ALB^8,9^, such that clearance of this parasite from the small intestine may also have contributed to the observed shifts. Although *T. trichiura* commonly resides in the ascending colon and cecum, these effects cannot be disentangled within the present study design and should be considered when interpreting the results.

From a taxonomic perspective, we did not observe high-level changes, such as shifts in the relative abundance of phyla or the prevalence of core microbial species. However, comparisons of taxonomic profiles between BL and FU at the specieslevel revealed significant associations across all treatment arms. Our findings align with existing literature, which describes the taxonomically broad yet species-specific antibacterial properties of both IVM and MOX that are also concentration-dependent^27,28^. A key pharmacological difference between the two anthelmintics lies in their administration: MOX is administered at a fixed dose of 8 mg, whereas IVM dosing is weight-based (200 µg/kg). According to Maier et al. estimated intestinal IVM concentrations for participants of this trial range from 1.8 µM to 7.2 µM and according to recent in vitro data, IVM inhibits growth of several gut-bacterial isolates in concentrations ≥ 5 µM^28^. Indeed, the stratification of the IVM/ALB cohort by absolute administered dose - into IVM/ALB-low (3–12 mg) and IVM/ALB-high (15–21 mg) arms - revealed a markedly greater extent of off-target effects in the IVM/ALB-high arm. This stratification introduced the risk of age bias; however, the age ranges were similar between arms (IVM/ALB-low: 12–58 years; IVM/ALB-high: 19–57 years), and baseline composite microbiome metrics did not differ significantly. Therefore, increased total amount of drug administered, and consequently increased intestinal drug concentrations, likely account for the observed off-target effects in the IVM/ALB-high arm. This interpretation is further supported by data from the IVM/ALB-low arm, where the taxonomic signals associated with treatment were comparably sparse. Currently, there is no data for estimated intestinal concentrations of MOX, but despite its lower molecular weight (IVM: 875.1 g/mol, MOX: 639.8 g/mol), we argue that the intestinal concentrations of MOX in the MOX/ALB cohort are lower compared to IVM in the IVM/ALB-high cohort given the difference in administered dose. This likely explains the sparsity in detected off-target effects in the MOX/ALB arm, despite seemingly stronger inhibitory effects of MOX on bacterial isolates in vitro^28^. In this respect, the MOX/ALB arm resembled the IVM/ALB-low arm and contrasted with the IVM/ALB-high arm.

This trend becomes even more pronounced at the functional level and is consistently confirmed across both MetaCyc pathway and reaction levels. First, ordination analyses revealed significant differences between timepoints in the IVM/ALB cohort, while no such effect was observed for the MOX/ALB cohort. Notably, within the IVM/ALB-high arm, a substantially larger proportion of variance was explained by timepoint, both at the level of individual reactions or broader metabolic pathways. In the IVM/ALB-high arm, the observed shifts in ordination centroids are likely driven by increased beta dispersion. This suggests that functional shifts are primarily occurring within individual participants, rather than reflecting a uniform, community-wide shift. Supporting this interpretation, pairwise comparison of reaction and pathway abundance and prevalence between BL and FU revealed widespread modulation across numerous functional domains in response to IVM/ALB-high treatment, whereas no comparable effects were detected in the IVM/ALB-low or MOX/ALB arms. The modulation of functional profiles in the IVM/ALB-high arm indicates substantial remodeling of community structure (as observed by taxonomy) and metabolism, characterized by the excessive proliferation and elimination of specific microbial species, as well as alterations in the synthesis and degradation of cellular structures and metabolites, with particular focus on peptidoglycan biosynthesis. Noteworthy, several modulated pathways associated with IVM/ALB-high treatment were recently linked to *T. trichiura* infection status^41^, which indicates a potential overlap between off-target treatment effects and infection-associated microbiome alterations. Interestingly, the trend of pronounced functional modulation did not clearly extend to ARGs, as we did not detect significant associations between timepoint and ARG abundance across treatment arms. This suggests that the gut resistome remained comparatively stable in response to treatment, despite some small and consistent shifts in individual features.

In contrast to the macrolides IVM and MOX, current literature does not conclusively report antibacterial properties for ALB. Previous studies investigating off-target effects of single-dose ALB in a cohort of 32 hookworm-infected individuals from Ghana linked successful treatment to significant structural and compositional alterations in the gut microbiota^42^. Notably, such taxonomic changes were absent in individuals where treatment failed. These findings suggest that observed microbiota shifts are more likely attributable to microbial rearrangements following helminth clearance, rather than direct effects of ALB itself. This interpretation is further supported by existing evidence indicating strong associations between helminth infection status and gut microbiota composition^41,43,44^. In our study, however, this issue cannot be fully disentangled. Although cure rates for *Trichuris* spp. infection were low (ALB: 13.4%, IVM/ALB: 22.5%, MOX/ALB: 15.3%), co-infections with *A. lumbricoides* were common (ALB: 35.7%, IVM/ALB: 46.5%, MOX/ALB: 32.9%)^33^. This remains relevant because *A. lumbricoides* inhabits the small intestine, whereas *Trichuris* spp. reside in the large intestine, which also harbors the highest density of the gut microbiota. Off-target parasite clearance may therefore still have contributed to the observed microbiota shifts, even if the ecological overlap with the bacterial community is likely greater for *Trichuris* spp. In addition, because ALB has reported activity against other microeukaryotes, including some protozoa and fungi, indirect effects mediated through non-bacterial members of the gut microbiome also cannot be excluded^45^. While we observed multiple enriched and depleted taxonomic features in the ALB-treated cohort, the functional profiles revealed only minimal differences between timepoints, confined to a limited set of metabolic functions (e.g., EC 1.14.14.1) and pathways. Furthermore, although ordination analyses detected significant taxonomic and functional shifts, the proportion of variance explained by timepoint was low. This indicates that the observed centroid shifts likely result from weak, diffuse community-wide changes rather than robust functional reprogramming. As we did not detect consistent taxonomic or functional signatures specific to ALB across treatment arms, these findings should be interpreted cautiously. Still, some direct or indirect ALB-related microbiota modulation cannot be excluded, and ALB may also have contributed to the effects observed in the IVM/ALB and MOX/ALB cohorts.

Despite the added value of our findings, we acknowledge several limitations of this study. First, our results are derived from a limited geographic location—7 villages in Côte d’Ivoire—while *Trichuris* spp. infections are widespread in the Global South. Moreover, having increased longitudinal sampling points, especially long-term follow-ups at 6–12 months, might help to further refine the off-target effects associated with anthelmintic exposure. This would help distinguish between transient and persistent effects. Another limitation is that detailed prior MDA histories were not available for the individual study districts, Dabou and Jacqueville. National WHO and ESPEN data indicate that preventive chemotherapy programmes for STH, lymphatic filariasis, onchocerciasis and trachoma were active in Côte d’Ivoire in 2021, making prior community exposure to IVM, ALB and, potentially, azithromycin plausible^46–50^. However, the available data are reported at national level and do not resolve the exact prior exposure history in our specific study communities. Nonetheless, prior exposure may therefore have influenced the baseline microbiome and the magnitude of the observed treatment effects.

In summary, we demonstrate that MOX/ALB, as well as IVM/ALB at lower concentration, do not induce substantial off-target alterations in the gut microbiota. Similarly, ALB-associated microbiota shifts appear limited in scope and likely represent indirect effects or unsystematic species turnover rather than direct and biologically meaningful community remodeling. In contrast, higher amounts of IVM ( ≥ 15 mg) were associated with pronounced taxonomic and functional perturbations, underscoring a concentration-dependent relationship in off-target effects, parallel with antibiotic exposure. These findings suggest that previously reported antibacterial properties of macrocyclic lactones may be more nuanced and context-dependent than generally assumed, influenced by dosage, treatment regimens, and host-specific factors. From a public health perspective, these results are especially relevant given the increasing interest in using higher-dose IVM regimens in malaria vector control programs^34^ and in treating STH infections^51^, especially when targeting adult populations. While such strategies may enhance antiparasitic efficacy or transmission interruption, our findings highlight the need to consider potential microbiota-mediated consequences, which could impact gut health, immunity and pathogen resistance dynamics in treated communities.

## Methods

### Sample origin

Stool samples were gathered as part of a randomized controlled trial (NCT04726969; https://clinicaltrials.gov/study/NCT04726969) aimed at evaluating the efficacy and safety of MOX/ALB compared to ALB and IVM/ALB against *T. trichiura* in Côte d’Ivoire in 2021^33^ (Fig. 1). The results of the trial were published by Sprecher et al.^33^. Individuals interested in participating in the trial were invited to complete the process of informed consenting including consent for the planned microbiome and pharmacogenomic analyses. Thereafter, consented individuals were assessed for study eligibility during screening procedures. Overall, 210 eligible participants were randomly assigned to three treatment arms, namely ALB (*n* = 67), IVM/ALB (*n* = 71) and MOX/ALB (*n* = 72). For each participant, a small aliquot of stool (approximately 1 g) was transferred to a sterile 2 ml cryotube, both before the treatment at baseline (BL) and 2–3 weeks after the treatment at follow-up (FU). The collected aliquots were immediately frozen and kept at −20 °C. Upon completion of the trial, these aliquots were shipped to Swiss TPH (Allschwil, Switzerland) on dry ice and kept at −80 °C until DNA extraction. Only fully anonymized samples and isolates were used throughout the study, and no participant-identifying information was available. The sequencing analysis was limited to 204 paired samples, i.e., ALB (*n* = 67), IVM/ALB (*n* = 69) and MOX/ALB (*n* = 68), for which BL and FU samples were available, and sequencing libraries could be produced. Since only IVM treatment is weight-based (200 µg/kg bodyweight), we further stratified the IVM/ALB arm according to the absolute dose of IVM received, measured by the number of 3 mg tablets administered. According to Maier et al., oral administration of 11.4 mg of IVM, which corresponds to an average amount of IVM following administration of 200 µg/kg used for STH infections, result in an estimated intestinal IVM concentration of 4.6 μM^27^. Moreover, previously published in vitro studies have demonstrated that a diverse range of gut-bacterial isolates exhibit pronounced sensitivity to IVM at concentrations of ≥ 5 μM^28^. Therefore, we separated the initial IVM/ALB cohort into a low-absolute dose group (IVM/ALB-low, 3–12 mg or 1–4 tablets, *n* = 44) and a high-absolute dose group (IVM/ALB-high, 15–21 mg or 5–7 tablets, *n* = 25). These groups will be referred to as arms, even though they did not represent a treatment arm of the trial per se (Supplementary Table 1).

### DNA extraction of frozen stool samples

To extract DNA from frozen stool samples, we used the DNeasy PowerSoil Pro kit (QIAGEN, 47016) and followed the manufacturer’s standard protocol using 100-150 mg of frozen stool. Final DNA concentrations were determined using a QuBit 4 fluorometer (Thermo Fisher Scientific, Q33238) with the 1x dsDNA HS Assay kit (Thermo Fisher Scientific, Q33231). Eluates were stored at −20 °C until further use.

### Illumina and Nanopore sequencing

DNA eluates were adjusted to a concentration of 15–30 ng/µl in nuclease-free water (Ultrapure™ Distilled water, Invitrogen, USA) prior to library preparation. Libraries were prepared using the NEBNext Ultra FS II (E7805L) reagents. Sequencing was performed on an S4 flow cell using an Illumina NovaSeq 6000 instrument targeting 18 M paired-end reads per sample (2 × 150 bp). Six samples did not produce any libraries; therefore, six sample pairs were excluded from any further analyses (Fig. 1). Nanopore libraries were prepared using the Oxford Nanopore Technologies Native Barcoding Kit 96 (SQK-NBD114-96) according to the manufacturer’s instructions. The libraries were sequenced on an Oxford Nanopore Technologies PromethION 2 solo instrument equipped with PromethION flow cells (FLO-PRO114M, R10.4.1) for 72 h with a target of 3−5 Gb sequenced bases per sample, according to Bertrand et al. (avg. Q-score > 14, avg. reads over Q20 > 79%).

### Overview of bioinformatics workflows

We applied two complementary bioinformatic workflows. First, a conventional workflow based on Illumina data was used to analyze taxonomic, functional and resistome profiles in paired BL and FU samples from each participant. Second, a workflow operating on the pooled data for the individual treatment arms was applied. The second combines Illumina short-read and Nanopore long-read data to generate high-quality metagenome-assembled genomes (MAGs) representative of each treatment arm and timepoint combination. The pooled design is aimed at maximizing recovery of high-quality MAGs for each treatment arm and timepoint, rather than to reconstruct individual-specific microbiomes in participant-level design. Moreover, the design of this workflow enabled linking antimicrobial resistance genes (ARGs) to their corresponding microbial host with annotated taxonomy. A comprehensive overview of the workflows is provided in Supplementary Fig. 1, and an abbreviated version is shown in Fig. 2.

### Preprocessing of short sequencing reads

Raw Illumina reads were processed with Trimmomatic^52^ (v. 0.39) for adapter removal and quality. Subsequently, non-bacterial reads were removed using Kraken2^53^ (v. 2.1.3) equipped with the “MinusB” RefSeq index (as of October 9, 2023), retaining unclassified reads. These processed Illumina reads were used in both the participant-level short-read workflow and the pooled treatment arm-level workflow.

### Nanopore read processing (Pooled treatment arm-level workflow)

Basecalling of the POD5 files obtained during Nanopore sequencing was performed using Dorado^54^ (v. 0.7.2) with the model dna_r10.4.1_e8.2_400bps_sup@v4.3.0. We then used Filtlong^55^ (v. 0.2.1) to remove reads shorter than 300 bp, supplying the processed paired-end Illumina reads as a filtering template. Adapters were subsequently removed using Dorado’s trimming function.

### Pooling and subsetting (Pooled treatment arm-level workflow)

To prepare the long-read data for pooled assembly, Nanopore reads from each sample were subsampled to 1 Gb (if available) using seqkit^56^ (v. 2.6.1) because Nanopore sequencing depth varied substantially between samples, ranging from 0.7 Gb to 14 Gb. This step ensured a more even contribution of long-read data from each participant to the pooled assemblies. The resulting Nanopore subsets were then pooled by treatment arm and timepoint combination. Processed Illumina reads were also pooled by treatment arm and timepoint, but no subsampling was required because Illumina sequencing depth was comparatively uniform across samples (Fig. 3A). To assess reproducibility of the MAG assembly process, each pool (comprising both Nanopore and Illumina reads) was split into three non-overlapping subsets using seqkit. This resulted in 24 pooled assembly datasets used for MAG reconstruction.

### Hybrid metagenome assembly (Pooled treatment arm-level workflow)

A first assembly iteration was done using SPAdes^57^ (v. 4.0.0) on short-read data. The resulting assemblies were further refined with the corresponding Nanopore long-read data using OPERA-MS^58^ (v. 0.9.0), equipped with the GTDB database^59^ (release 89, June 17, 2019).

### Binning, refinement and MAG quality control (Pooled treatment arm-level workflow)

Using the contigs generated by OPERA-MS, we applied multiple binning algorithms, including MetaBAT2^60^ (v. 2.15), MaxBin2^61^ (v. 2.2.7), and CONCOCT^62^ (v. 1.1.0), and combined the results using DAS Tool^63^ (v. 1.1.7). The resulting bins were assessed with CheckM2^64^ and only bins with completeness above 95% and contamination below 5% were retained for downstream analyses.

### MAG taxonomic annotation (Pooled treatment arm-level workflow)

To maintain taxonomic consistency between the resulting MAGs and the results from the participant-level workflow, the MAGs were taxonomically annotated using MetaPhlAn4^65^. For the purpose of taxonomic annotation, a custom Python script was used to fragment each MAG into 150 bp sequences and used as input for MetaPhlAn4. MAGs with ambiguous or missing annotations were excluded from further analysis. As an independent validation step, we additionally annotated MAGs using BAT^66^ (v. 6.0.1; Supplementary Table “SUPP_BAT_vs_MP4”). Because BAT applies stringent assignment criteria, concordance between the two approaches could primarily be assessed at higher taxonomic levels.

### Combining MAGs of condition subsets (Pooled treatment arm-level workflow)

To dereplicate MAGs recovered from the three subsampled assemblies of each treatment arm and timepoint condition, species-level MetaPhlAn annotations were combined with CheckM2 quality reports using a custom Python script. All unique species-level MAGs were retained, whereas for replicated MAGs only the most complete representative, defined by the highest CheckM2 completeness, was kept for further analysis (Supplementary Fig. 25).

### Phylogenetic analysis of MAGs (Pooled treatment arm-level workflow)

For each treatment arm, we constructed phylogenetic trees including MAGs recovered from both BL and FU using CheckM^67^. As a quality control measure, we added selected core genomes identified in this study from the MetaPhlAn database (CHOCOPhlAn database v. Oct22). The resulting alignment file was used as input for iqtree2^68^ for tree building and bootstrapping. Trees were plotted in R-Studio using the packages ape^69^ (v. 5.8-1), ggtree^70^ (v. 3.14.0), and ggtreeExtra (v. 1.16.0).

### Functional profiling and analysis (Pooled treatment arm-level workflow)

To compare the functional content of pooled MAGs between treatment arms and timepoints, MAGs were annotated using Bakta^71^ (v. 1.9.3). Enzyme commission (EC) annotations were merged with a custom Python script and plotted in R-Studio using the same visualization packages described below for taxonomic profiling.

### Resistome profiling and analysis (Pooled treatment arm-level workflow)

To assign ARGs to specific microbial hosts and compare changes in ARG abundance between BL and FU with changes in the abundance of the corresponding taxa, we characterized the resistome at the MAG level (Fig. 6). ARGs were first identified in MAGs using the Resistance Gene Identifier equipped with the CARD database^72^. For each MAG, a bowtie2^73^ (v. 2.5.2) reference index containing its identified ARGs was then generated. Processed Illumina reads from all samples were aligned to these MAG-specific indices, and samtools (v. 1.19.2) with the idxstats command was used to compute absolute mapped read counts for each index. Counts were converted to relative counts normalized by gene count per million gene reads and gene length. To compare fold changes in MAG-specific ARG abundance between BL and FU with fold changes of the corresponding host taxa, only MAGs with unambiguous MetaPhlAn-based taxonomic annotation were retained, and only taxa detected at both timepoints were included in the analysis.

### Taxonomic profiling (Participant-level workflow)

For participant-level taxonomic profiling, we used MetaPhlAn (v. 4.0.6) equipped with the CHOCOPhlAn database (v. Oct22) to compute taxonomic relative abundances for each sample from quality-controlled Illumina reads. For subsequent analyses, we applied a 1% prevalence filter and a 20% variance filter in R-Studio (R-base v. 4.4.1) using dplyr^74^ (v. 1.1.4).

### Diversity and community structure (Participant-level workflow)

Alpha-diversity metrics were then calculated from the filtered relative abundance table. Richness, Shannon diversity (H′), Berger-Parker dominance (BP), here corresponding to the relative abundance of the most abundant taxon. Spearman correlations for these metrics between BL and FU were determined in R-Studio using the packages car^75^ (v. 3.1-2), plyr^76^ (v. 1.8.9), and vegan^77^ (v. 2.6-6.1). Phylum-level relative abundance barplots were also generated in R, additionally using tidyr^78^ (v. 1.3.1). Bray-Curtis dissimilarities and non-metric multidimensional scaling (NMDS), together with PERMANOVA and PERMDISP analyses, were also computed using vegan (v. 2.6-6.1). The core microbiome was defined using the microbiome^79^ (v. 1.28.0), phyloseq^80^ (v. 1.50.0), and reshape2^81^ (v. 1.4.4) packages.

### Differential abundance analysis (Participant-level workflow)

For differential abundance analysis of taxonomic features between BL and FU within each treatment arm, we used MaAsLin3^82^ (v. 0.99.1) together with Random Forest analyses using the randomForest^83^ (v. 4.7-1.2) package. For MaAsLin3, associations were tested separately within each treatment arm using a linear mixed effects model with timepoint as a fixed effect and participant ID as a random intercept. Following the authors’ recommendations, the unfiltered relative abundance table was used as input, together with total sum scaling (TSS) normalization and logarithmic (LOG) transformation.

### Functional profiling and analysis (Participant-level workflow)

Functional profiles were generated for each sample using HUMAnN4^84^ (v. 4.0.0.alpha.1) equipped with the CHOCOPhlAn database (v. Oct22), with quality-controlled paired-end Illumina reads used as input. We then generated unstratified tables via human_split_stratified_table and converted absolute counts to relative abundances with human_renorm_table. This was performed for both MetaCyc pathways (based on the pathabundance output) and MetaCyc reactions (based on the genefamilies output). Both relative abundance tables were subjected to a 10% prevalence filter. Bray-Curtis dissimilarity, NMDS, PERMANOVA, and PERMDISP analyses were subsequently performed as described above. Differential abundance analysis between BL and FU within each treatment arm was performed with MaAsLin3 using the model described above. For clarity of presentation, and to facilitate interpretation of the large number of associations identified, the results were additionally filtered by retaining only the 20% lowest and 20% highest coefficients for pathways (Supplementary Fig. 21) and the 5% lowest and 5% highest coefficients for reactions (Supplementary Fig. 23).

### Resistome profiling and analysis (Participant-level workflow)

Using the Illumina read files, we performed participant-specific resistome characterization using bowtie2 (v. 2.5.2) equipped with a reference index based on the Comprehensive Antibiotic Resistance Database (CARD, v. 3.3.0). Samtools (v. 1.19.2) with the idxstats command was then used to compute the absolute number of mapped reads for each sample and reference ARG. Absolute counts were converted to relative counts normalized by gene count per million gene reads. The resulting relative abundance table was subjected to a 1% prevalence filter. ARG beta-diversity analyses and differential abundance analyses between BL and FU within each treatment arm were then performed using MaAsLin3 as described above.

### Visualization

For all visualizations, the following R packages were used as appropriate: RColorBrewer (v. 1.1-3), ggplot2^85^ (v. 3.5.1), ggsignif^86^ (v. 0.6.4), gridExtra (v. 2.3), ggpubr^87^ (v. 0.6.0), ggtext^88^ (v. 0.1.2), ggVennDiagram^89^ (v. 1.5.2) and cowplot^89^ (v. 1.1.3).

### Statistics and reproducibility

Group comparisons were conducted using Mann-Whitney U test for unpaired two-group comparisons and Wilcoxon Signed Rank-Sum tests for comparisons involving two paired groups, and all *p*-values were adjusted using the Benjamini-Hochberg correction – if not stated otherwise. To compare pairwise abundance and prevalence of taxa, MetaCyc pathways, MetaCyc reactions and ARGs we employed MaAsLin3 as described previously. For the subsequent analysis, we utilized the individual and joint “q-vals” put forth by MaAsLin3, which were also corrected using the Benjamini-Hochberg procedure. Both for MaAsLin3 and the PERMANOVA/PERMDISP analyses, we employed a significance threshold of *p* < 0.1. We provided a detailed documentation of the bioinformatics analysis (pipeline and R-scripts) under https://github.com/STPHxBioinformatics/mac_data_analysis.

## Supplementary information

260423_REV_MAC_manuscript_SUPP

SUPP_BAT_vs_MP4

## Data Availability

The sequencing data (Nanopore and Illumina shotgun sequencing) generated in this study have been deposited in the NCBI Short Read Archive under the accession PRJNA1249224. The MAGs generated and used in this study have been deposited under 10.5281/zenodo.16262422^90^. Numerical source data underlying all figures, as well as detailed documentation of the bioinformatics analysis can be found under https://github.com/STPHxBioinformatics/mac_data_analysis.

## Code availability

The detailed documentation of the bioinformatics analysis can be found under https://github.com/STPHxBioinformatics/mac_data_analysis.

## Declaration on the use of AI

In the process of preparing this manuscript, AI-tools (ChatGPT) were leveraged for language editing, and grammatical correction. During preparation of the GitHub repository, AI-tools (ChatGPT) were utilized for code clean-up and code annotation (R-scripts). Hence, these tools solely served an editorial purpose and did not contribute to the study’s concept, analysis or conclusion. All scientific content in this manuscript represents the sole responsibility of the authors, who have thoroughly reviewed and approved the final versions of the text and illustrations.
