# Supplementary material for: Combined high-quality metagenomics reveals off-target effects of albendazole, ivermectin-albendazole and moxidectin-albendazole on the human gut bacteria
Source: NPJ Biofilms Microbiomes. 2026 May 29;12:165. doi: 10.1038/s41522-026-01018-3 (PMC13503709; doi:10.1038/s41522-026-01018-3)
Supplement: Supplementary file 1 [file 41522_2026_1018_MOESM1_ESM.pdf]

Supplementary Data concerning the manuscript:

Combined high-quality metagenomics reveals off-target effects of albendazole, ivermectin-albendazole and moxidectin-albendazole on the human gut bacteria.

Julian Dommann[a,b], Viviane P. Sprecher[a,b], Christian Beisel[c], Daniel Ballmer[a,b], Eveline Hürlimann[a,b], Jean T. Coulibaly[a,b,d,e], Jennifer Keiser[a,b], Pierre H. H. Schneeberger[a,b]*

[a]Swiss Tropical and Public Health Institute, Department of Medical Parasitology and Infection Biology, Allschwil, Switzerland.

[b]University of Basel, Basel, Switzerland.

[c]ETH Zürich, Department of Biosystems Science and Engineering, Basel, Switzerland

[d]Unité de Formation et de Recherche Biosciences, Université Félix Houphouët-Boigny, Abidjan, Côte d'Ivoire.

[e]Centre Suisse de Recherches Scientifiques en Côte d'Ivoire, Abidjan, Côte d'Ivoire.

* Correspondence to Pierre H. H. Schneeberger, pierre.schneeberger@swisstph.ch.

Supplementary Table 1: Study results summary, adapted from Sprecher *et. al.*[30]. *T. trichiura* infection intensity classification is based on World Health Organization criteria. Abbreviations: SD, standard deviation; CR, cure rate; ALB, albendazole; IVM/ALB, ivermectin-albendazole; MOX/ALB, moxidectin-albendazole.

**Characteristic**	**ALB**	**IVM/ALB**	**IVM/ALB-low**	**IVM/ALB-high**	**MOX/ALB**
Number of participants, n	67	69	44	25	68
Age [years]					
$Mean_{arit}$ (±SD)	26.9 ± 14.3	29.1 ± 15.5	23.8 ± 15.3	38.5 ± 10.6	30.0 ± 15.7
Range	12 to 58	12 to 58	12 to 58	19 to 57	12 to 60
Sex, n (%)					
Female	37 (55.2%)	39 (56.5%)	23 (52.3%)	16 (64.0%)	31 (45.6%)
Male	30 (44.8%)	30 (43.5%)	21 (47.7%)	9 (36.0%)	37 (54.4%)
Weight [kg]					
$Mean_{arit}$ (±SD)	59.0 ± 17.2	57.5 ± 17.8	47.0 ± 12.2	76.0 ± 8.7	56.4 ± 16.5
Range	23 to 100	23 to 95	23 to 65	65 to 95	28 to 104
Infection Intensity, n (%)					
light	53 (79.1%)	57 (82.6%)	34 (77.3%)	23 (92.0%)	53 (77.9%)
moderate	12 (17.9%)	10 (14.5%)	8 (18.2%)	2 (8.0%)	15 (22.1%)
heavy	2 (3.0%)	2 (2.9%)	2 (4.5%)	0 (0.0%)	0 (0.0%)
treatment efficacy					
CR (%)	13.4%	23.2%	20.5%	28.0%	13.2%

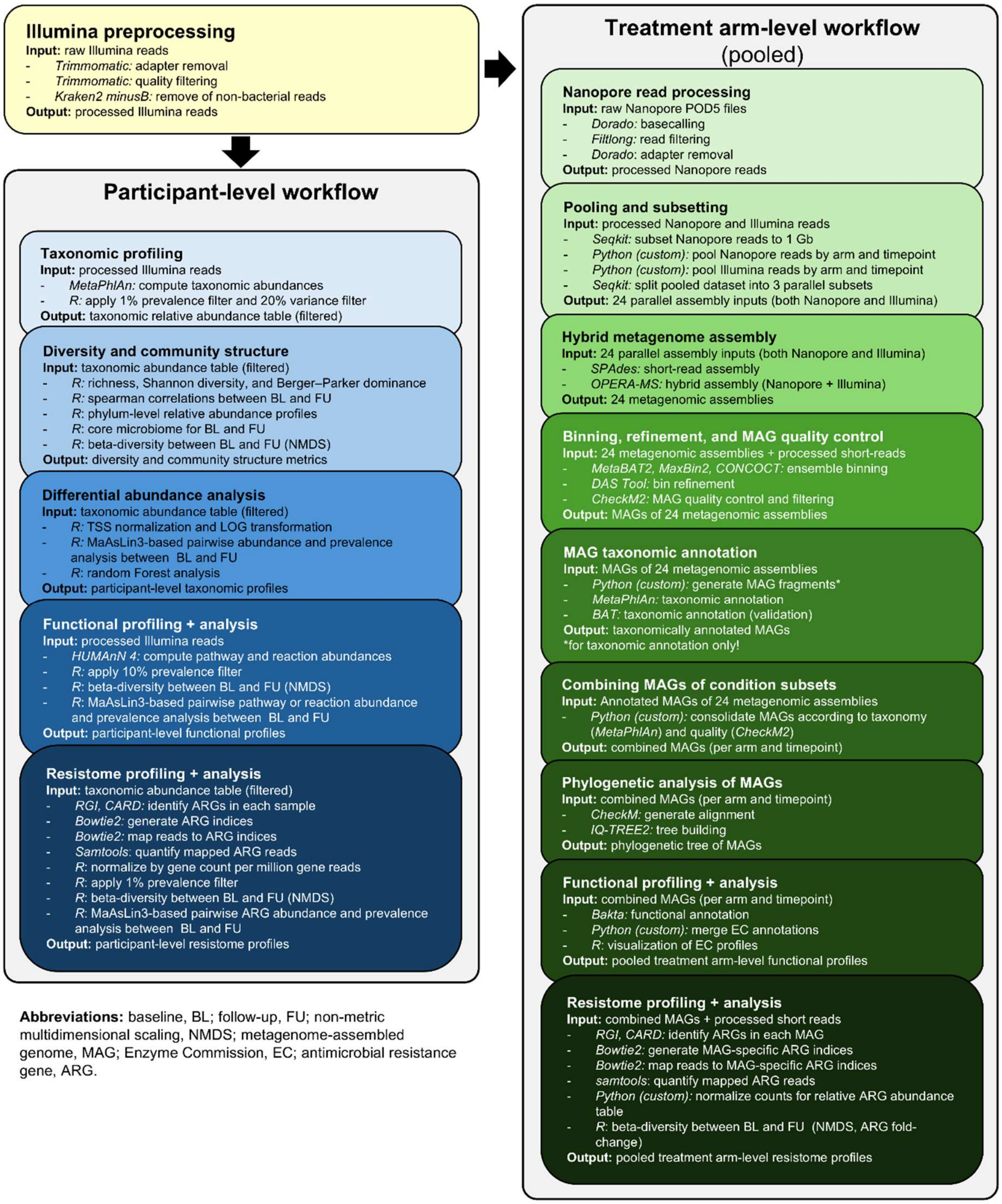

Supplementary Figure 1: Detailed overview of the bioinformatics pipeline utilized to process both short reads and long reads yielding taxonomic, functional and resistome profiles on participant- and pooled treatment-arm levels.

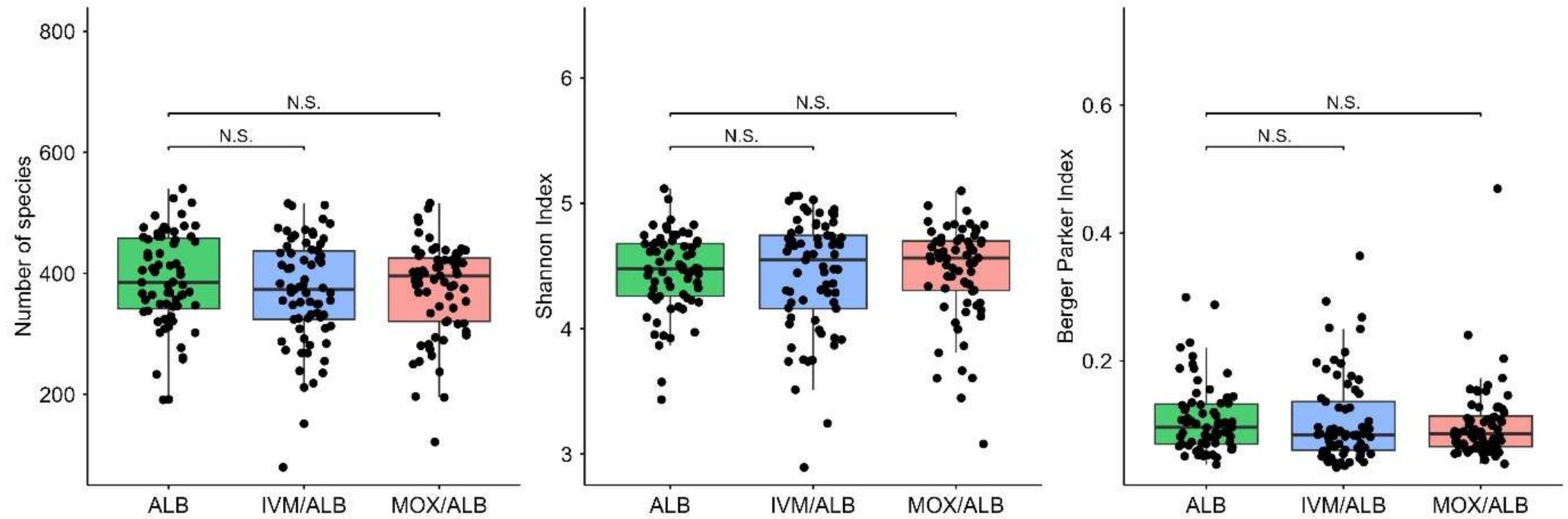

Supplementary Figure 2: Distributions of composite metrics richness, Shannon Index and Berger-Parker Index in the baseline cohort, stratified by treatment arm. We utilized a Wilcoxon Rank Sum test to assess statistical significance. Abbreviations: ALB, albendazole; IVM/ALB, ivermectin-albendazole; MOX/ALB, moxidectin-albendazole.

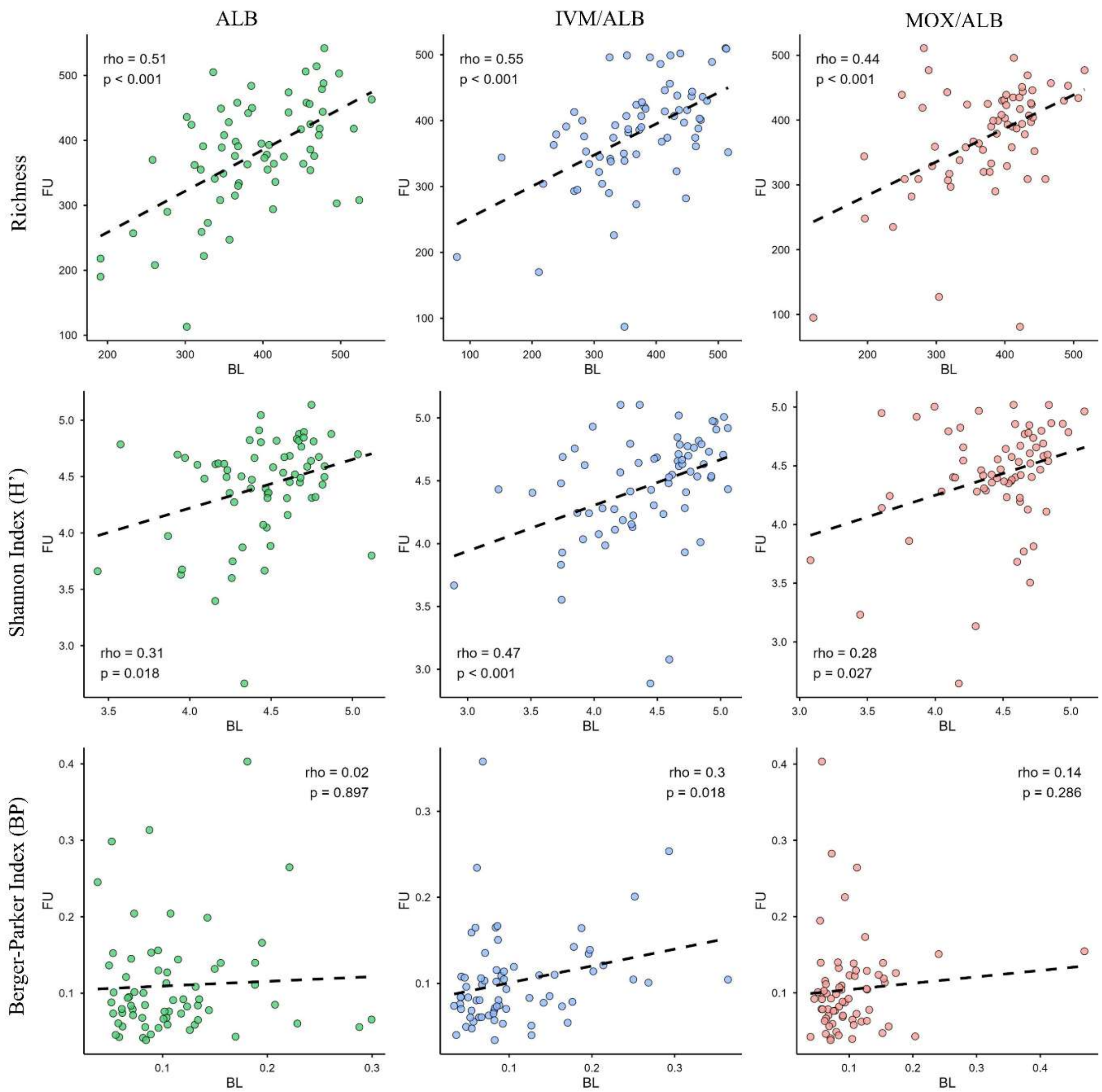

Supplementary Figure 3: Spearman correlations of the composite metrics Richness, Shannon Index and Berger-Parker Index for baseline (BL) and follow-up (FU) timepoints in each treatment arm. Abbreviations: ALB, albendazole; IVM/ALB, ivermectin-albendazole; MOX/ALB, moxidectin-albendazole.

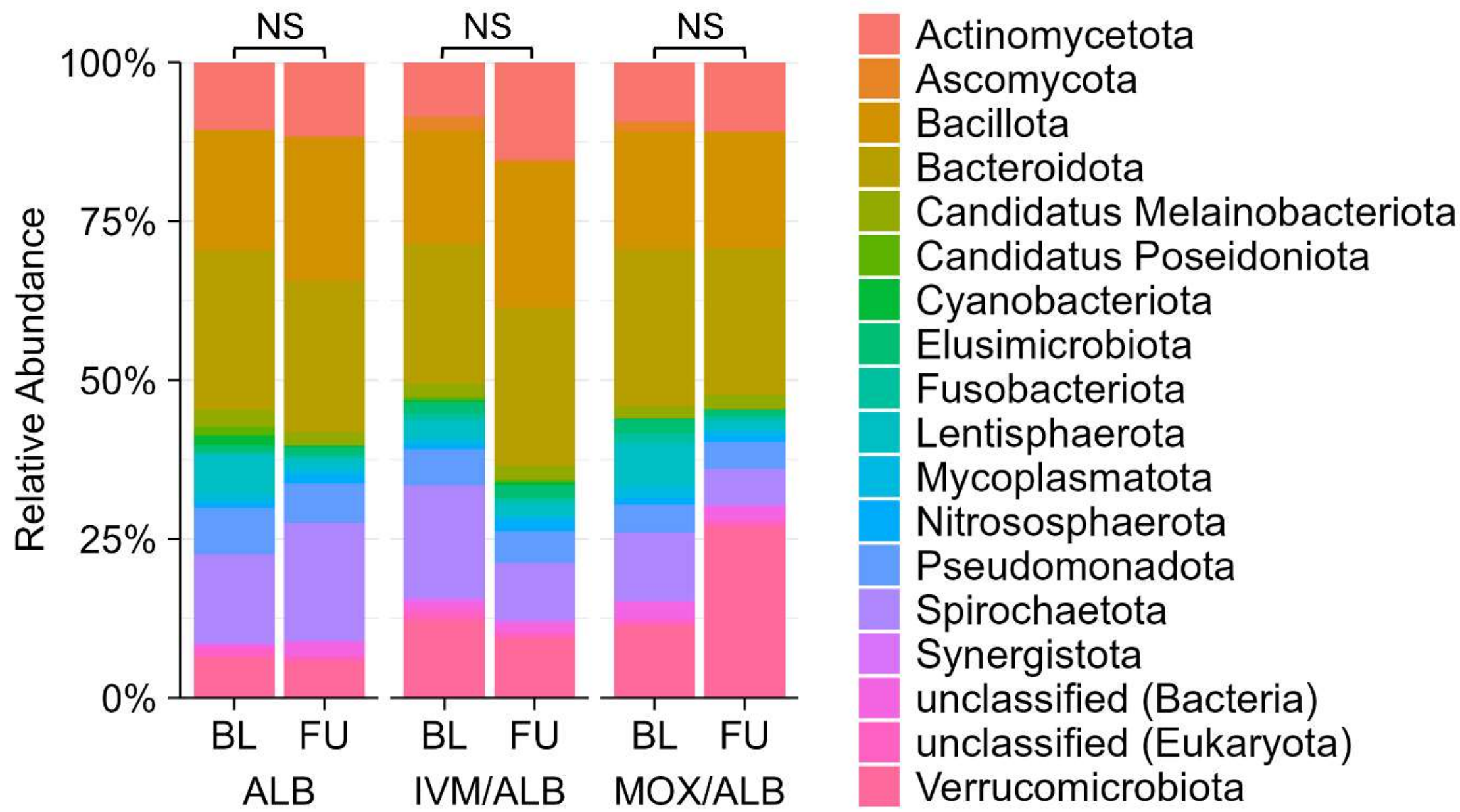

Supplementary Figure 4: Phylum-level relative abundances for baseline (BL) and follow-up (FU) timepoints across three treatment arms. Abbreviations: ALB, albendazole; IVM/ALB, ivermectin-albendazole; MOX/ALB, moxidectin-albendazole. Wilcoxon Signed-Rank Test (BH-corrected): $p = 0.832$ (ALB), 0.832 (IVM/ALB), 0.389 (MOX/ALB). Phylum nomenclature was adapted according to Oren *et. al.* (2021).

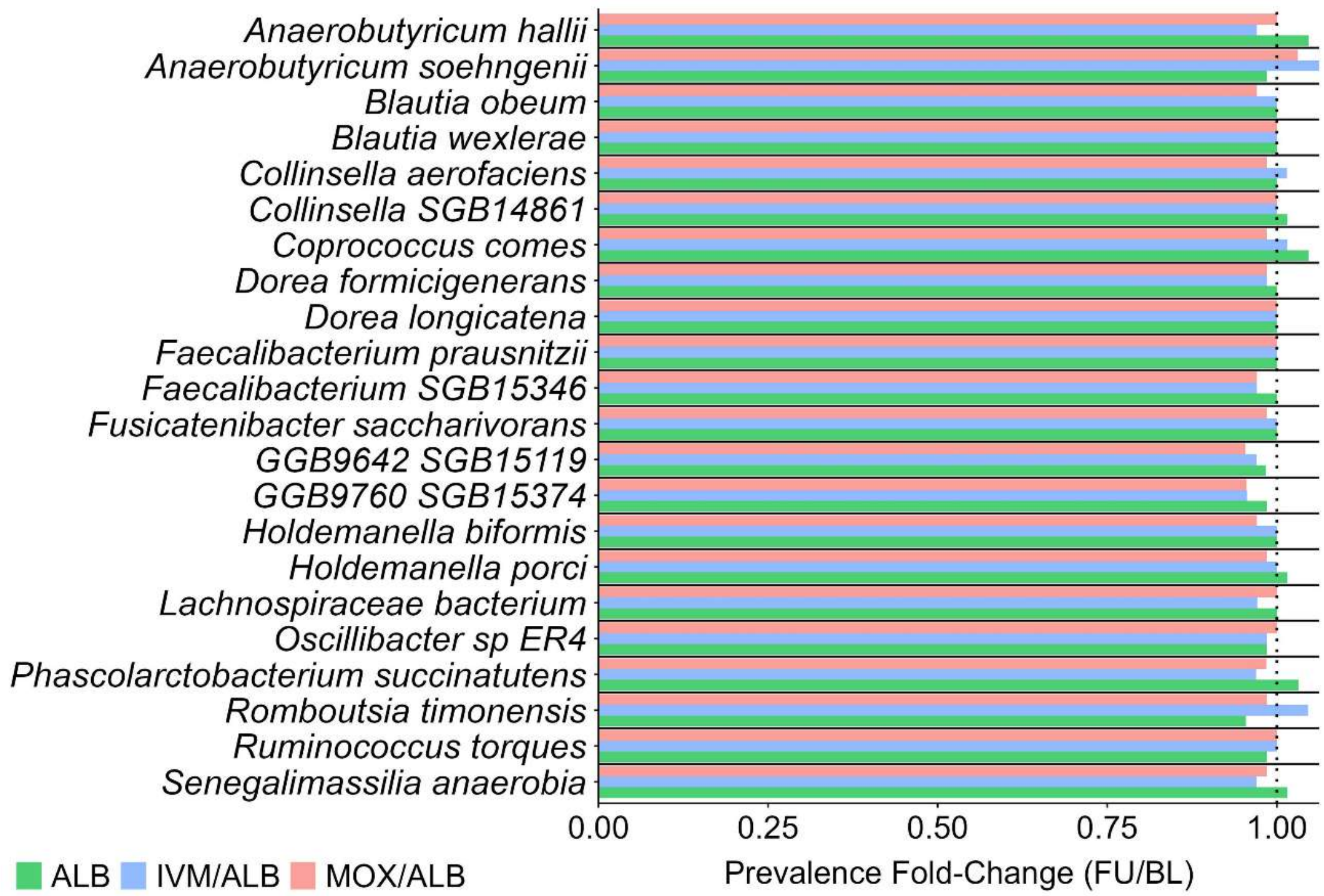

Supplementary Figure 5: Prevalence fold-change of core microbiota (FU/BL) for the three treatment arms ALB, IVM/ALB and MOX/ALB. Wilcoxon Rank-Sum Test (BH-corrected): p = 0.779 (ALB), 0.365 (IVM/ALB), 0.024 (MOX/ALB). Abbreviations: BL, baseline; FU, follow-up; ALB, albendazole; IVM/ALB, ivermectin-albendazole; MOX/ALB, moxidectin-albendazole.

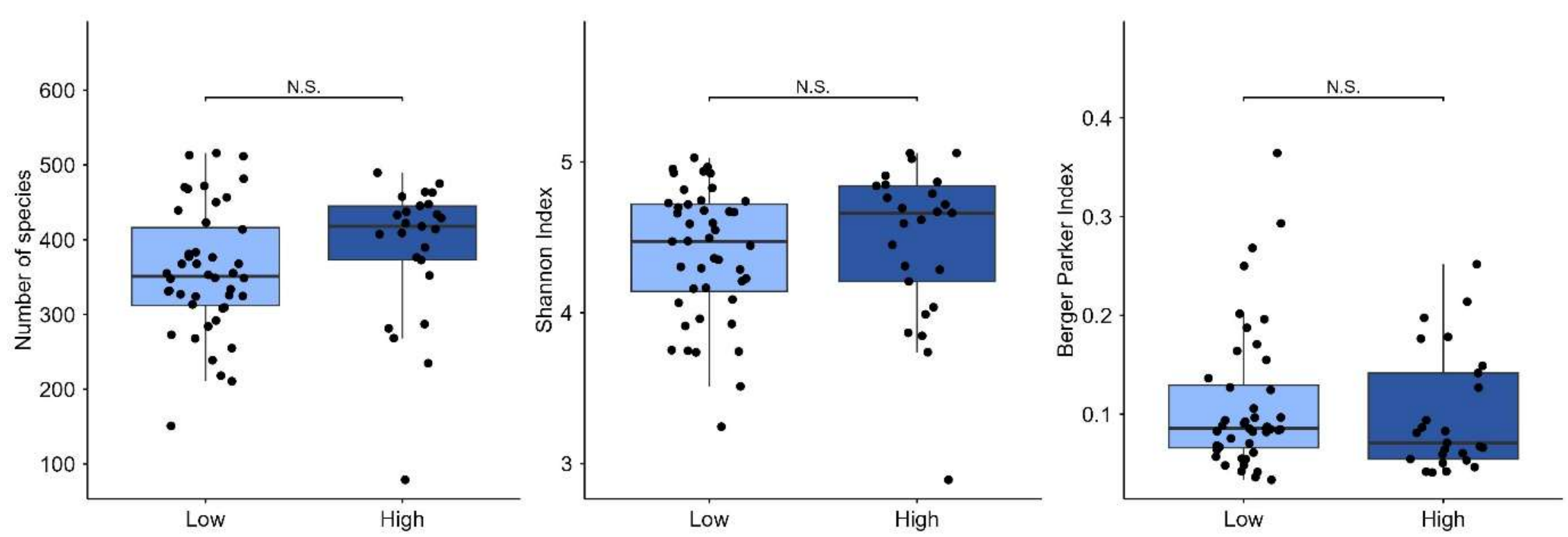

Supplementary Figure 6: Distributions of the composite metrics richness, Shannon Index and Berger-Parker Index in the baseline cohort of the IVM/ALB arm, stratified by dosage category. We utilized a Wilcoxon Rank Sum test to assess statistical significance.

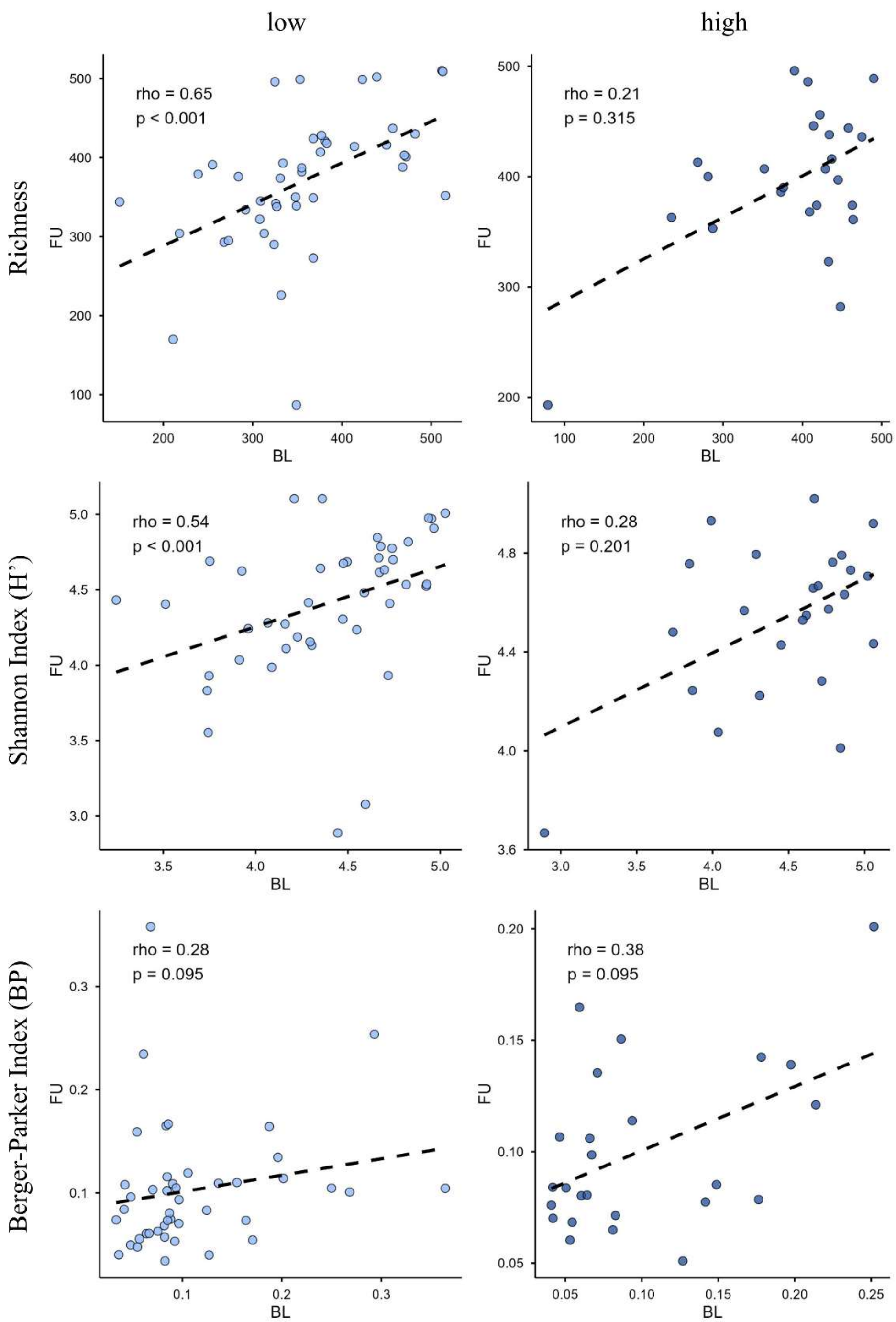

Supplementary Figure 7: Spearman correlations of the composite metrics richness, Shannon Index and Berger-Parker Index for baseline (BL) and follow-up (FU) timepoints in the two dosage categories of the IVM/ALB arm.

Supplementary Table 2: P-values of Mann-Whitney Test comparing baseline composite metrics (Shannon Diversity (H'), richness and Berger-Parker Index (BP)). Abbreviations: ALB, albendazole; IVM/ALB, ivermectin-albendazole; MOX/ALB, moxidectin-albendazole.

p-value (BH)	MOX/ALB vs. ALB	IVM/ALB vs. ALB	IVM/ALB-low vs. IVM/ALB-high
H'	0.5744	0.7839	0.5744
richness	0.5744	0.5744	0.5744
BP	0.5744	0.5744	0.5744

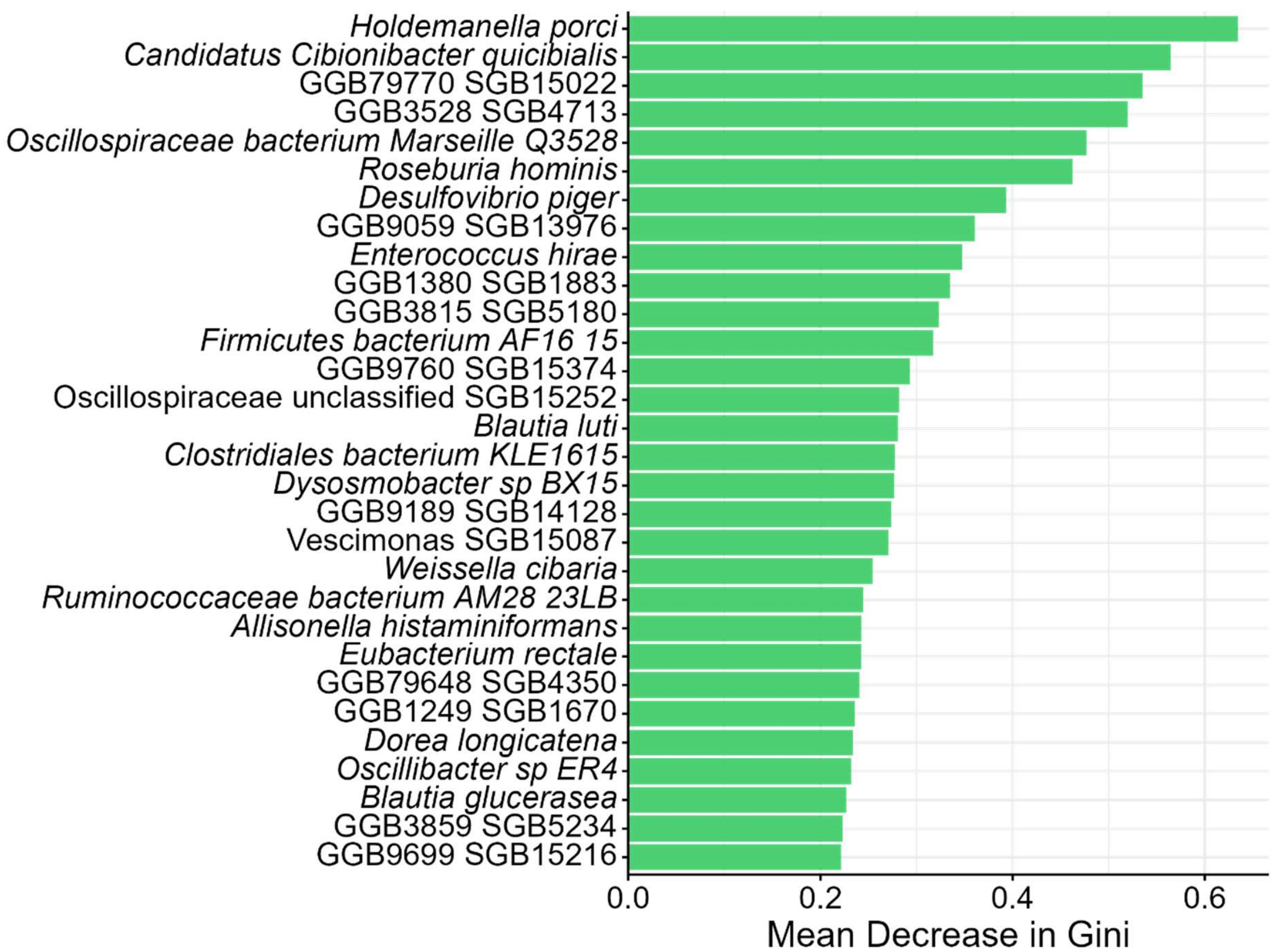

Supplementary Figure 8: Level of importance of bacterial species in separation of the baseline- and follow-up-associated taxonomic profiles in the albendazole arm by a Random Forest-based approach represented by the mean decrease in Gini. The species are listed in order of importance from top to bottom.

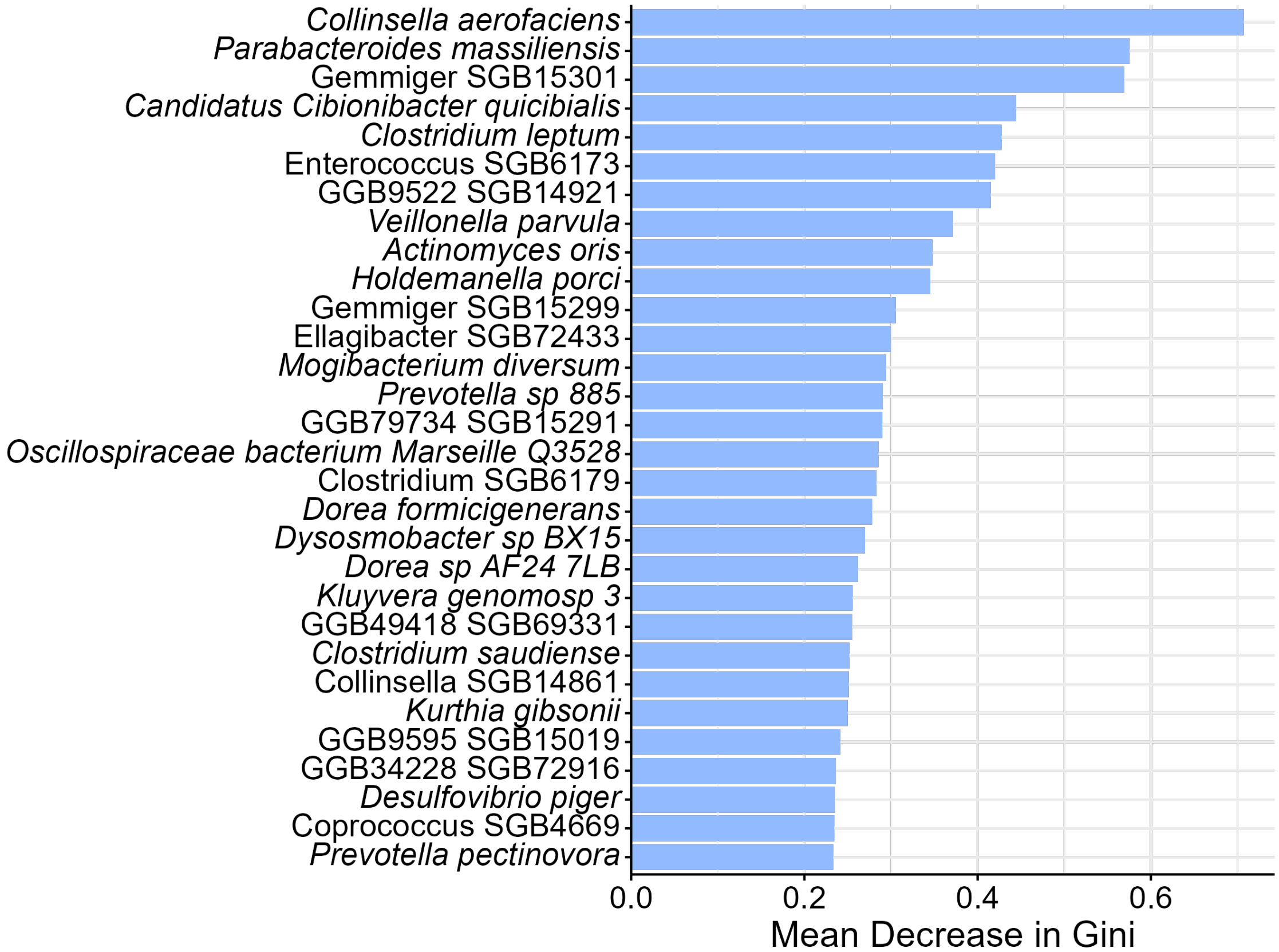

Supplementary Figure 9: Level of importance of bacterial species in separation of the baseline- and follow-up-associated taxonomic profiles in the ivermectin-albendazole arm by a Random Forest-based approach represented by the mean decrease in Gini. The species are listed in order of importance from top to bottom.

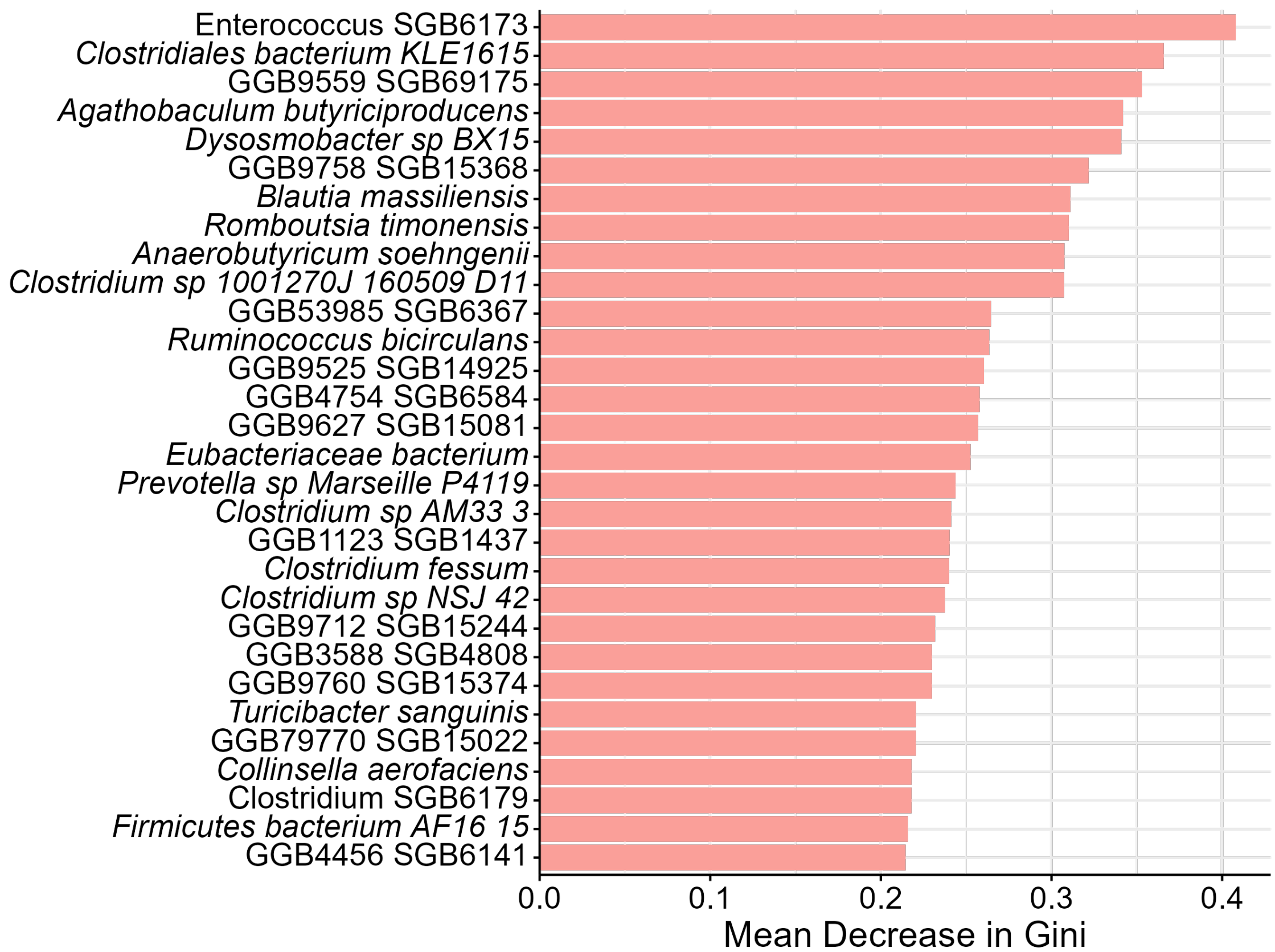

Supplementary Figure 10: Level of importance of bacterial species in separation of the baseline- and follow-up-associated taxonomic profiles in the moxidectin-albendzole arm by a Random Forest-based approach represented by the mean decrease in Gini. The species are listed in order of importance from top to bottom.

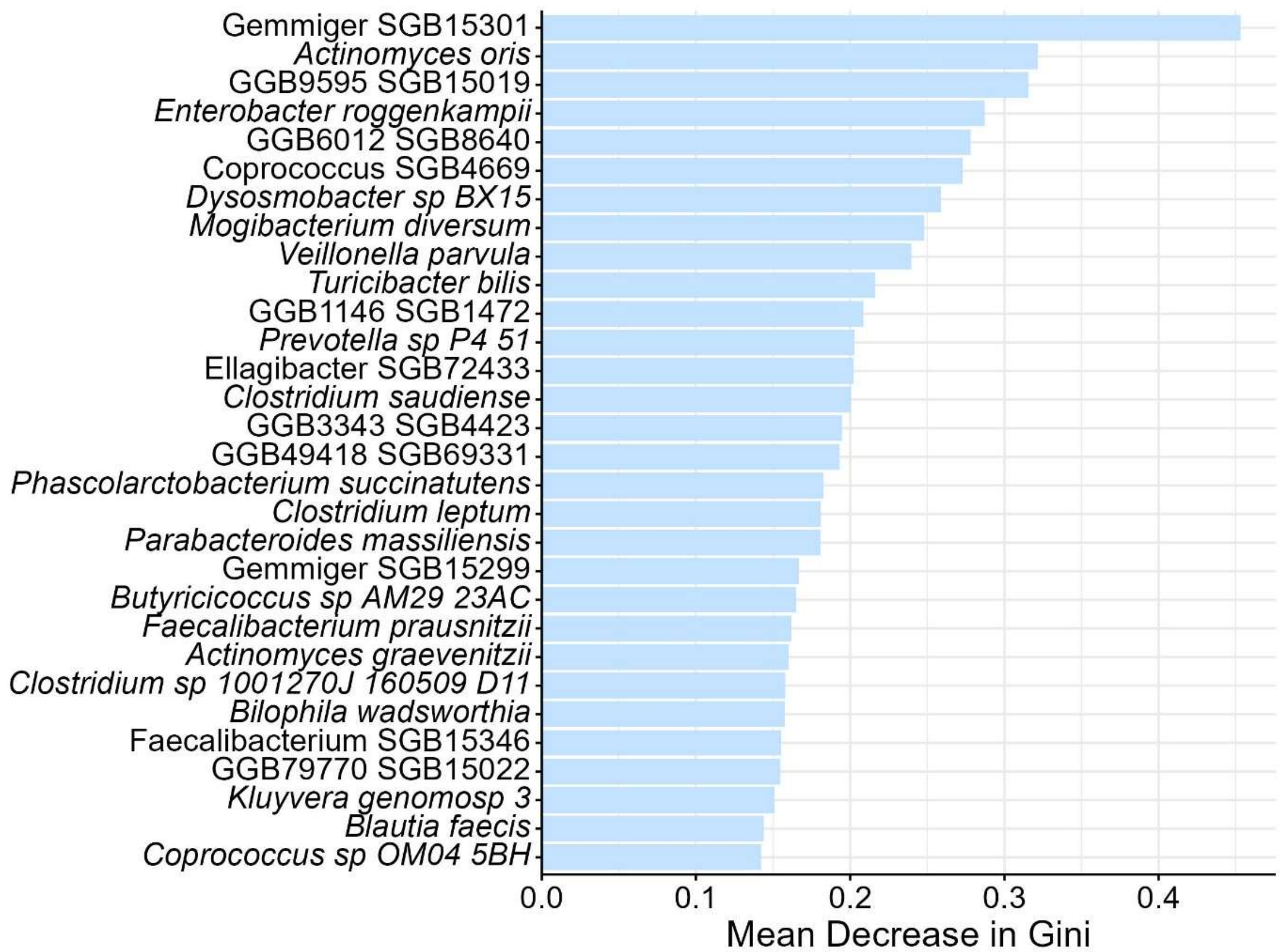

Supplementary Figure 11: Level of importance of bacterial species in separation of the baseline- and follow-up-associated taxonomic profiles in the "low" dosage category of the ivermectin-albendazole arm by a Random Forest-based approach represented by the mean decrease in Gini. The species are listed in order of importance from top to bottom.

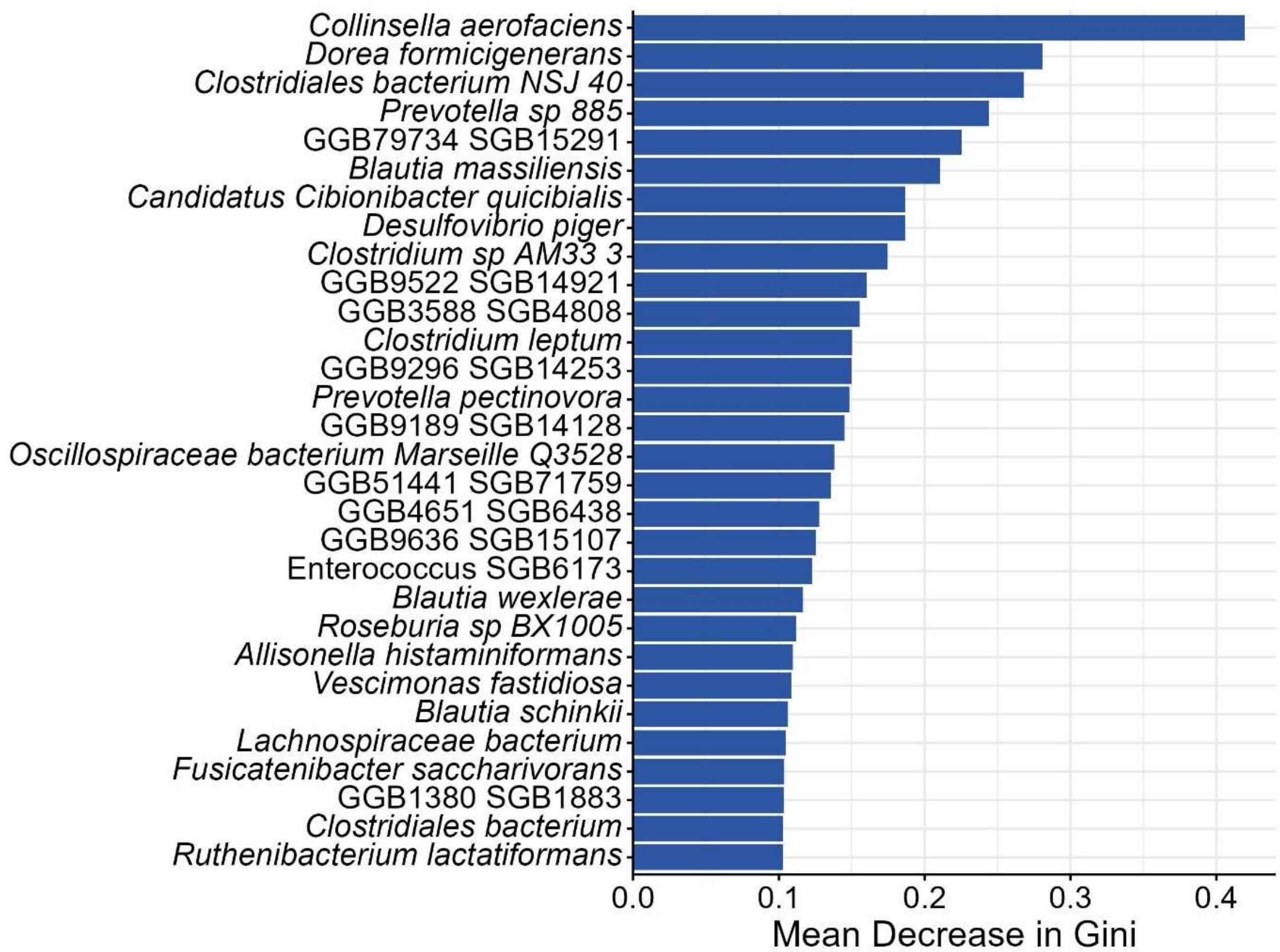

Supplementary Figure 12: Level of importance of bacterial species in separation of the baseline- and follow-up-associated taxonomic profiles in the "high" dosage category of the ivermectin-albendazole arm by a Random Forest-based approach represented by the mean decrease in Gini. The species are listed in order of importance from top to bottom.

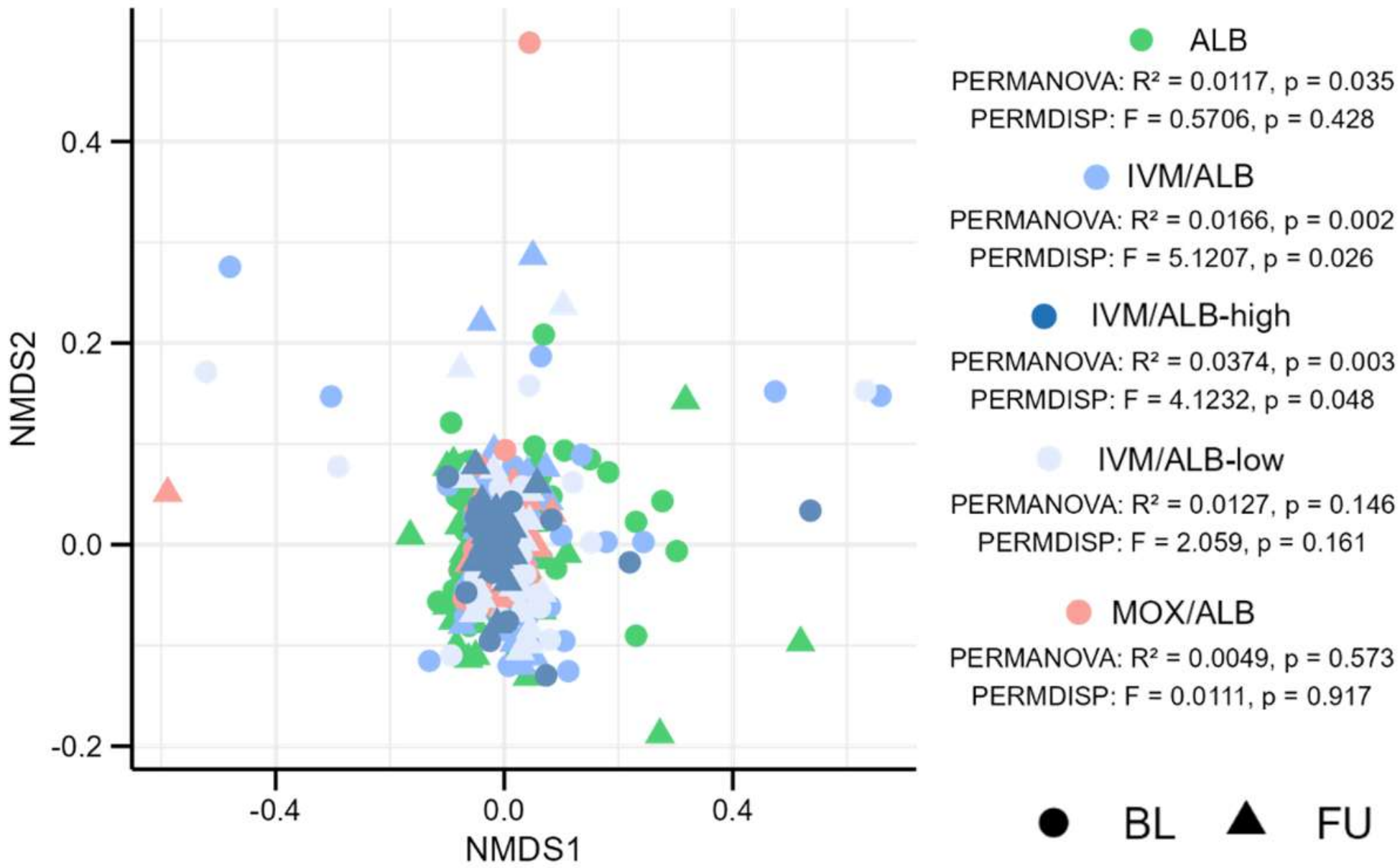

Supplementary Figure 13: Non-metric multidimensional scaling (NMDS) ordination based on Bray-Curtis Dissimilarity matrices of relative MetaCyc reaction abundances of paired baseline (BL) and follow-up (FU) samples. The stratification of the IVM/ALB is based on the absolute administered dose of IVM (IVM/ALB-low: 3-12mg, IVM/ALB-high: 15-21mg). PERMANOVA: p = 0.035 (ALB), 0.002 (IVM/ALB), 0.003 (IVM/ALB-high), 0.146 (IVM/ALB-low), 0.573 (MOX/ALB). PERMDISP: p = 0.428 (ALB), 0.026 (IVM/ALB), 0.048 (IVM/ALB-high), 0.161 (IVM/ALB-low), 0.917 (MOX/ALB). Abbreviations: ALB, albendazole; IVM/ALB, ivermectin-albendazole; MOX/ALB, moxidectin-albendazole.

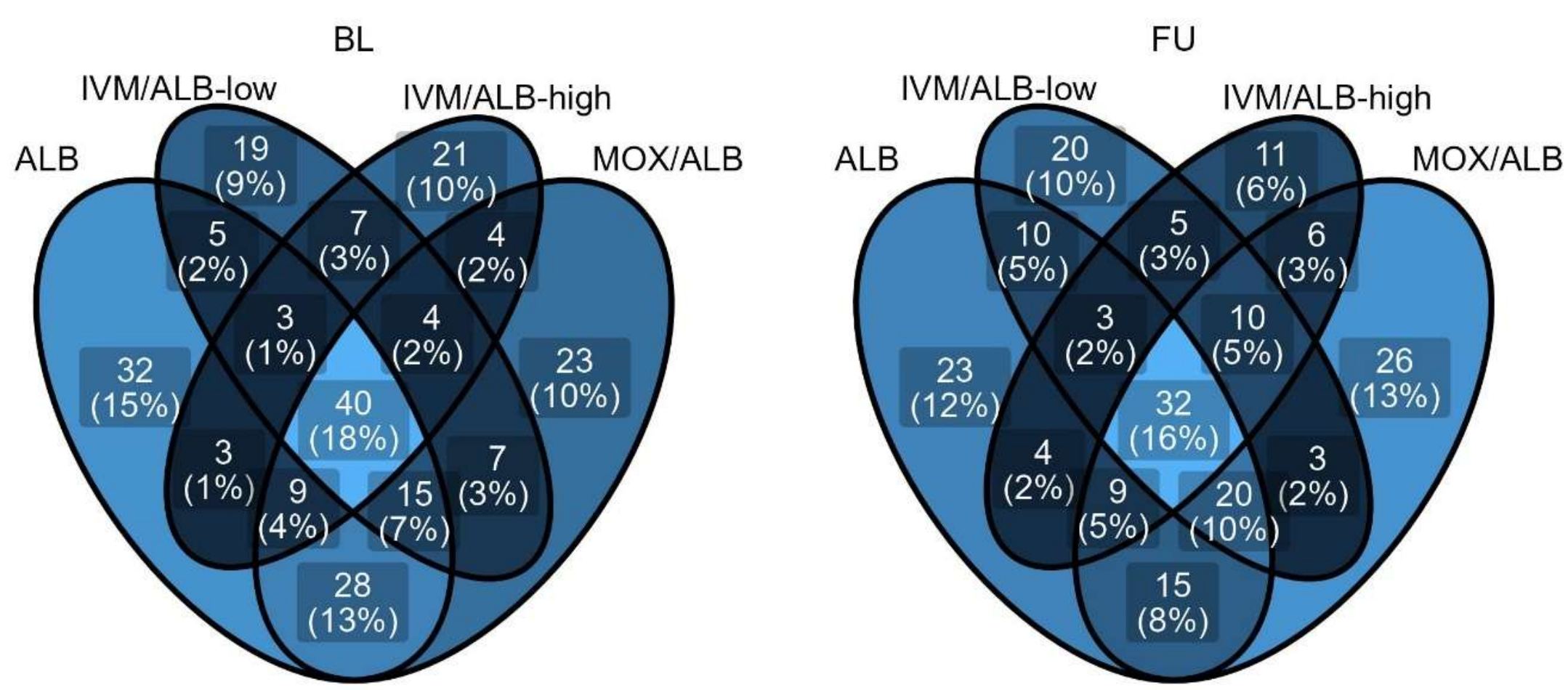

Supplementary Figure 14: Absolute number of shared and unique metagenomic bins between treatment arms (ALB, IVM/ALB-low, IVM/ALB-high, MOX/ALB). Abbreviations: BL, baseline; FU, follow-up; ALB, albendazole; IVM/ALB, ivermectin-albendazole; MOX/ALB, moxidectin-albendazole.

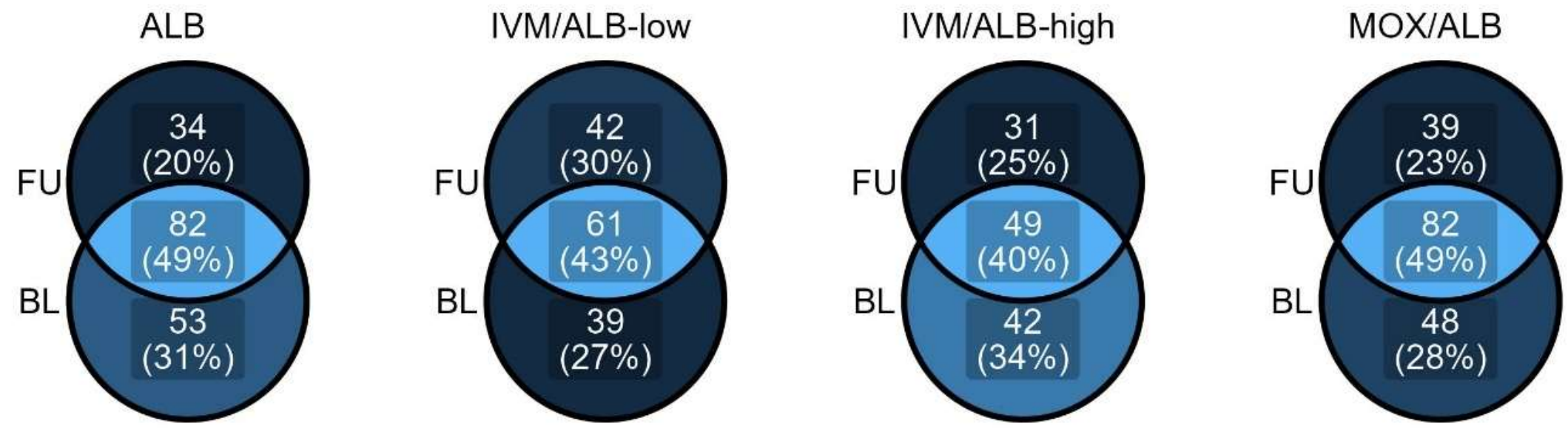

Supplementary Figure 15: Absolute number of shared and unique metagenomic bins between timepoints (BL, FU). Abbreviations: BL, baseline; FU, follow-up; ALB, albendazole; IVM/ALB, ivermectin-albendazole; MOX/ALB, moxidectin-albendazole.

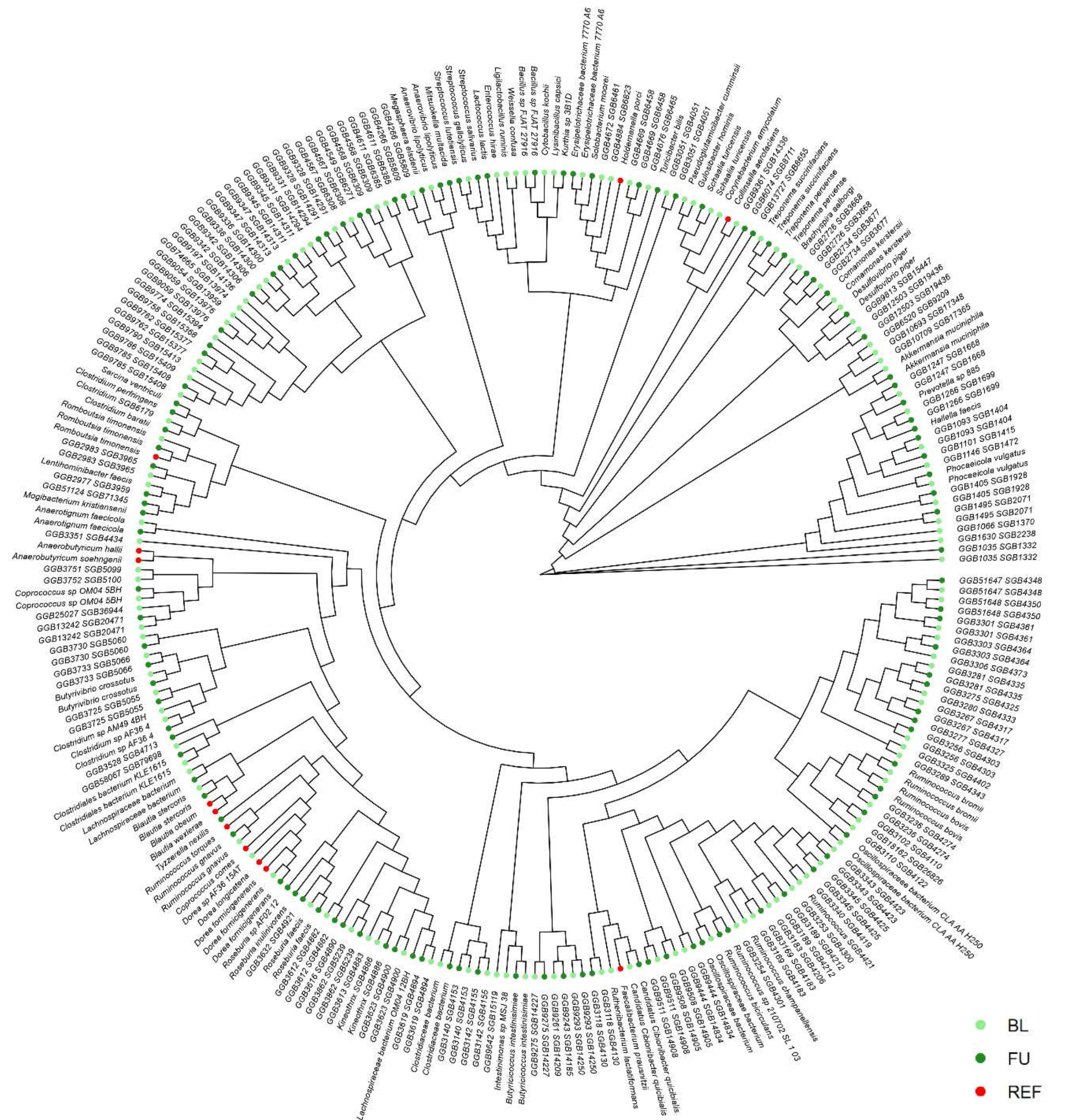

Supplementary Figure 16: Phylogenetic tree of the clean metagenomic assembled genomes (MAGs; Completeness > 95%, Contamination < 5%) of the albendazole cohort. MAGs are colored based on the timepoint (BL = baseline, FU = follow-up). Bootstrapping values (n = 1000) are indicated at the tree nodes. Reference genomes (REF) of the identified core species were obtained via the CHOCOPhlAn database (Jan 2021) and are colored in red.

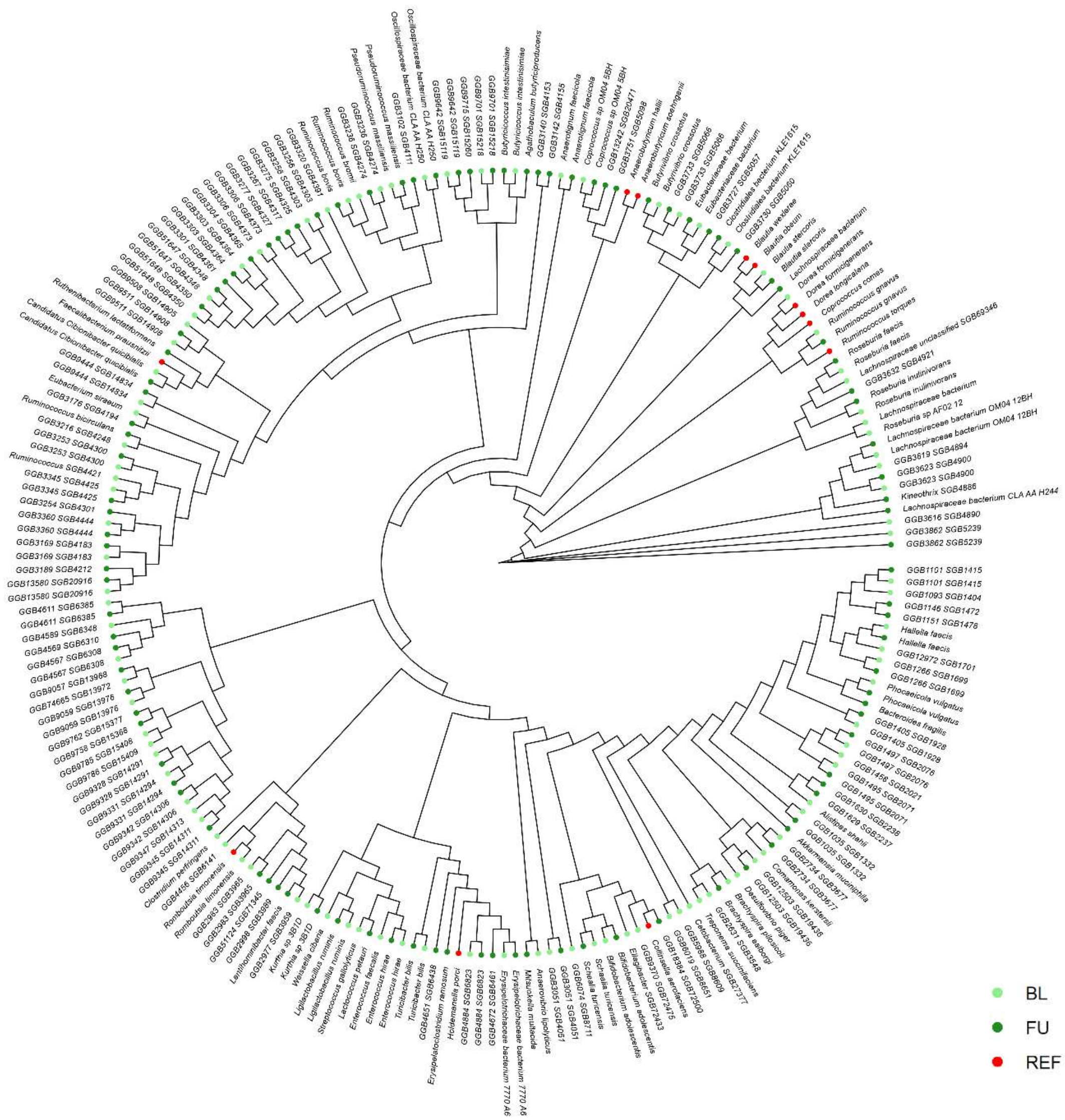

Supplementary Figure 17: Phylogenetic tree of the clean metagenomic assembled genomes (MAGs; Completeness > 95%, Contamination < 5%) of the ivermectin-albendazole-low cohort. MAGs are colored based on the timepoint (BL = baseline, FU = follow-up). Bootstrapping values (n = 1000) are indicated at the tree nodes. Reference genomes (REF) of the identified core species were obtained via the CHOCOPhlAn database (Jan 2021) and are colored in red.

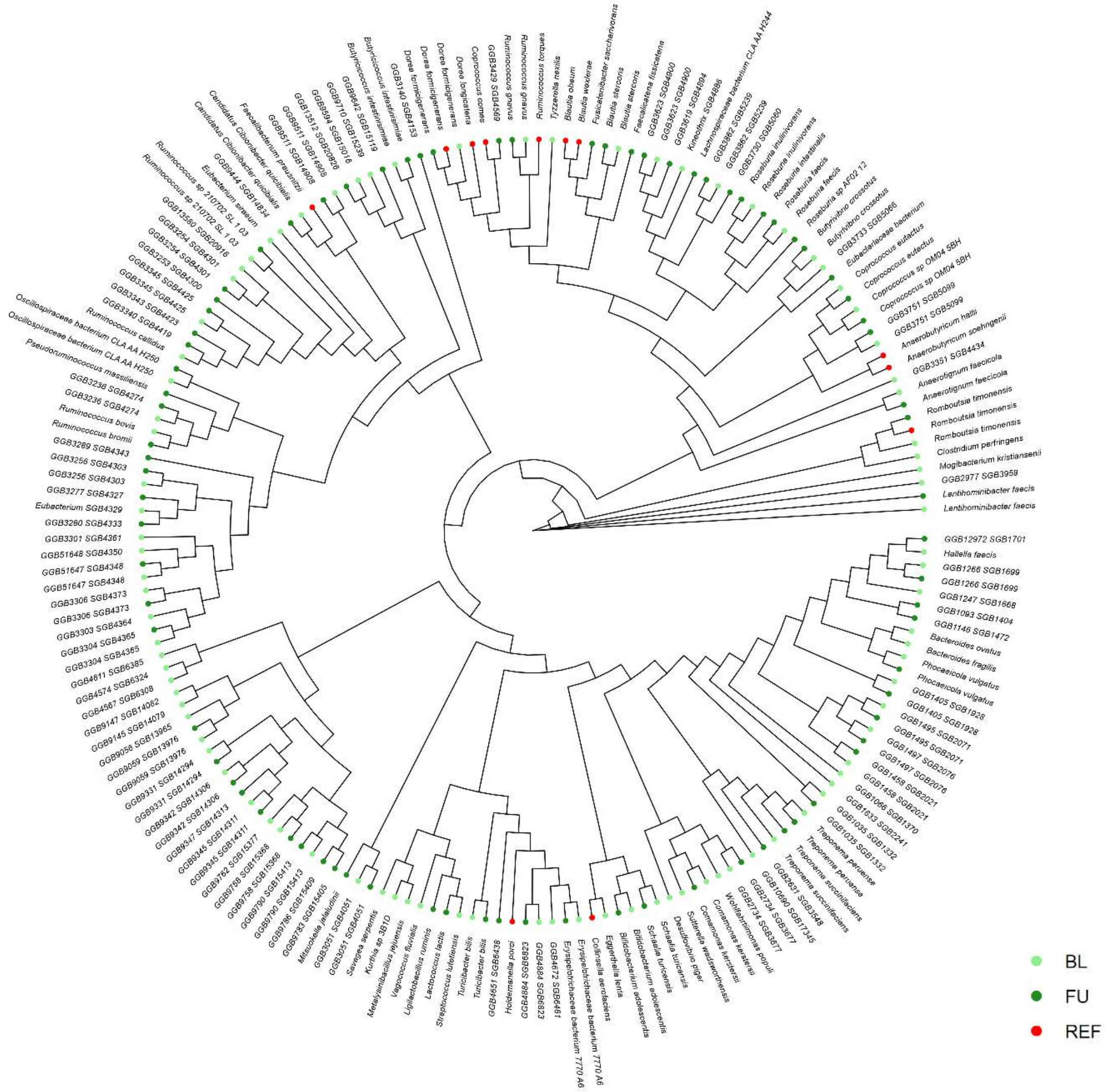

Supplementary Figure 18: Phylogenetic tree of the clean metagenomic assembled genomes (MAGs; Completeness > 95%, Contamination < 5%) of the ivermectin-albendazole-high cohort. MAGs are colored based on the timepoint (BL = baseline, FU = follow-up). Bootstrapping values (n = 1000) are indicated at the tree nodes. Reference genomes (REF) of the identified core species were obtained via the CHOCOPhlAn database (Jan 2021) and are colored in red.

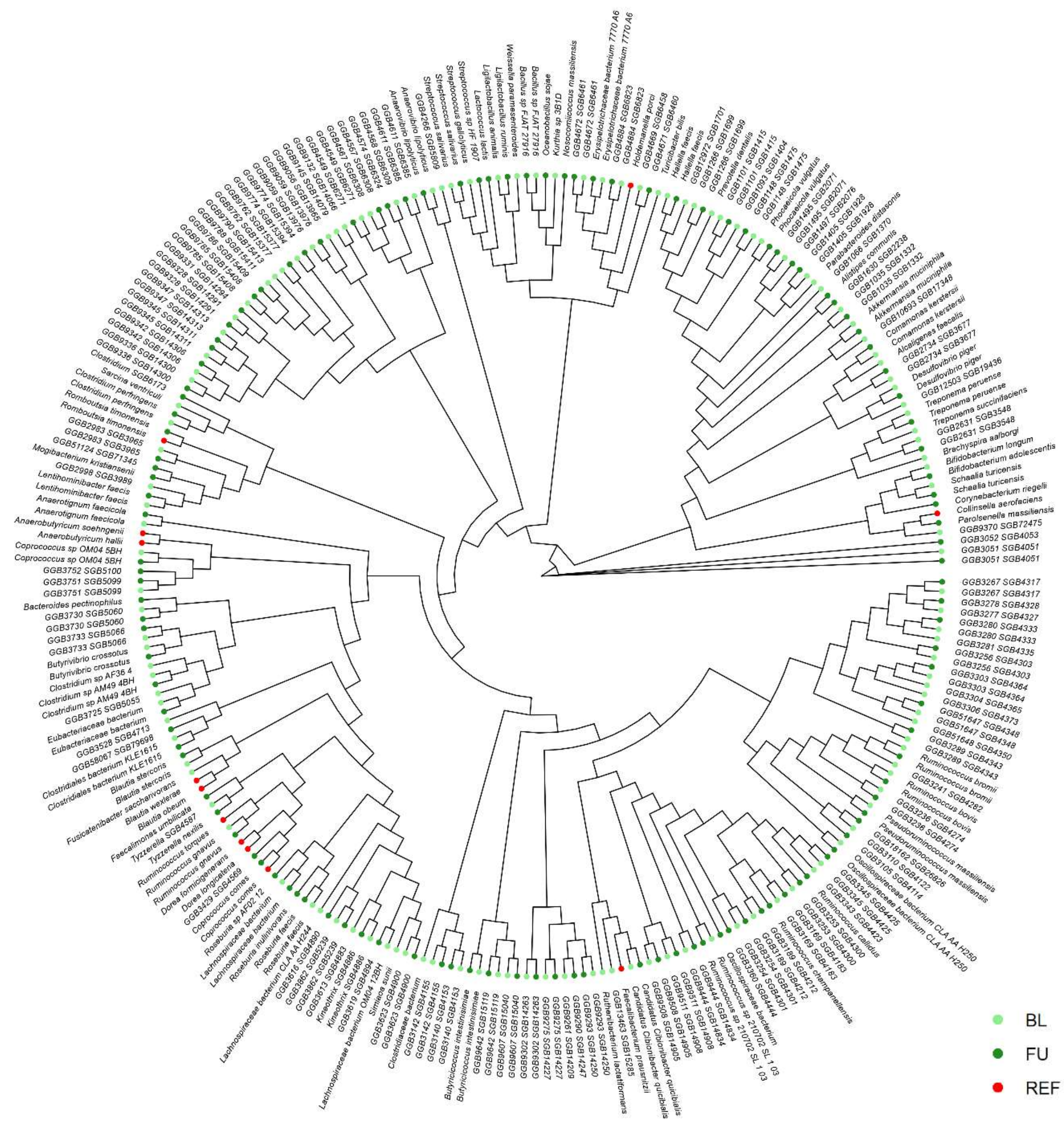

Supplementary Figure 19: Phylogenetic tree of the clean metagenomic assembled genomes (MAGs; Completeness > 95%, Contamination < 5%) of the moxidectin-albendazole cohorts. MAGs are colored based on the timepoint (BL = baseline, FU = follow-up). Bootstrapping values (n = 1000) are indicated at the tree nodes. Reference genomes (REF) of the identified core species were obtained via the CHOCOPhlAn database (Jan 2021) and are colored in red.

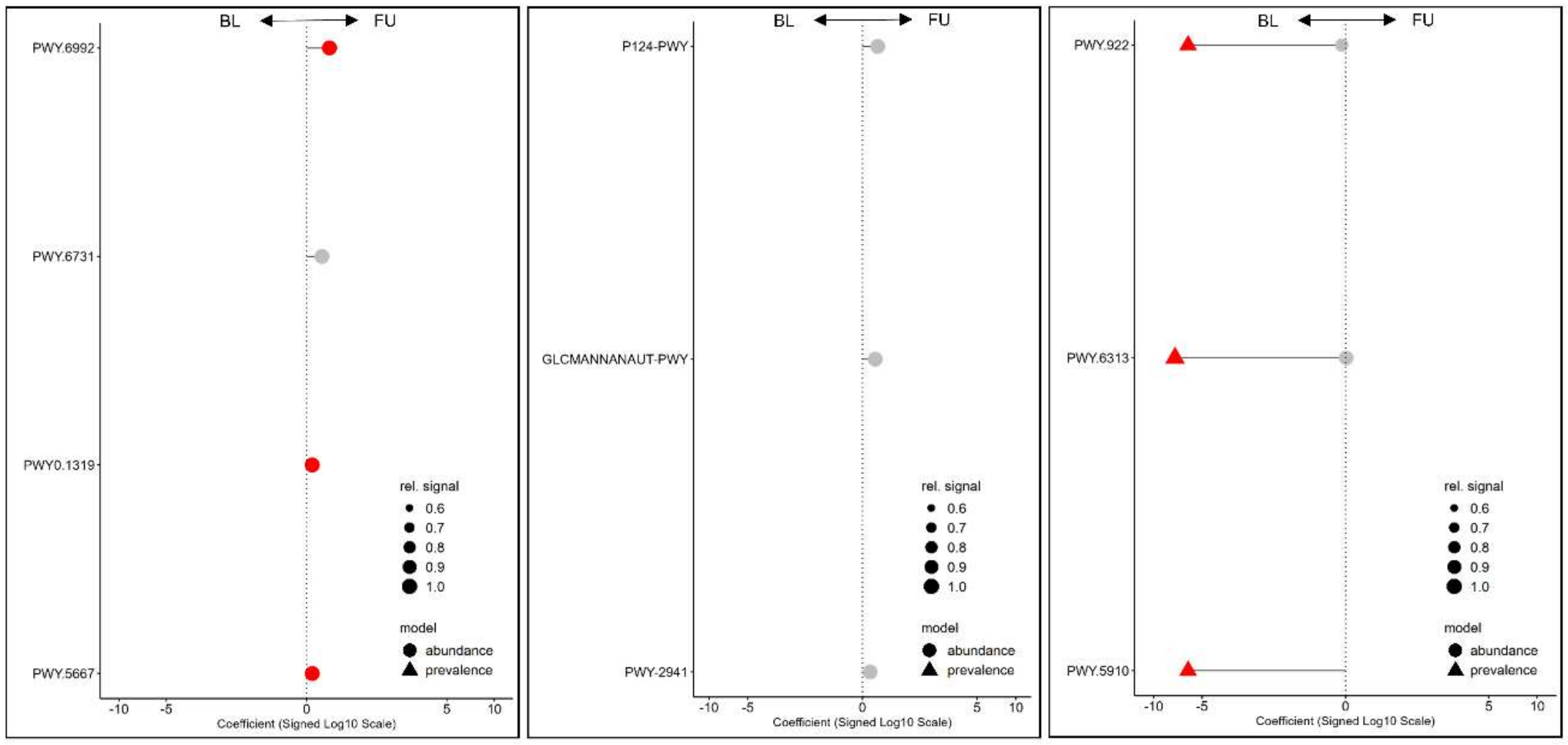

Supplementary Figure 20: Pairwise, species-level abundance and prevalence modelling of pathways between timepoints (BL = baseline, FU = follow-up) of ALB (**left**), IVM/ALB-low (**center**) and MOX/ALB (**right**) after filtering (keeping only the associations with the 20% lowest and 20% highest MaAsLin3 coefficients and a relative signal > 0.5). Signals to the left of the x-axis centerline indicate association with BL, signals to the left of the x-axis centerline indicate association with FU. The relative signal was calculated as the ratio of samples, in which the association was detected divided by the total number of samples for that arm and timepoint. Only pathways reaching a "joint q-val" of < 0.1 are shown. Pathways with "individual q-val" < 0.1 are depicted in red. Coefficients for the abundance models are given as $\log_2$(FU/BL). Coefficients for the prevalence models are given as ln(OR). Abbreviations: ALB, albendazole; IVM/ALB, ivermectin-albendazole; MOX/ALB, moxidectin-albendazole.

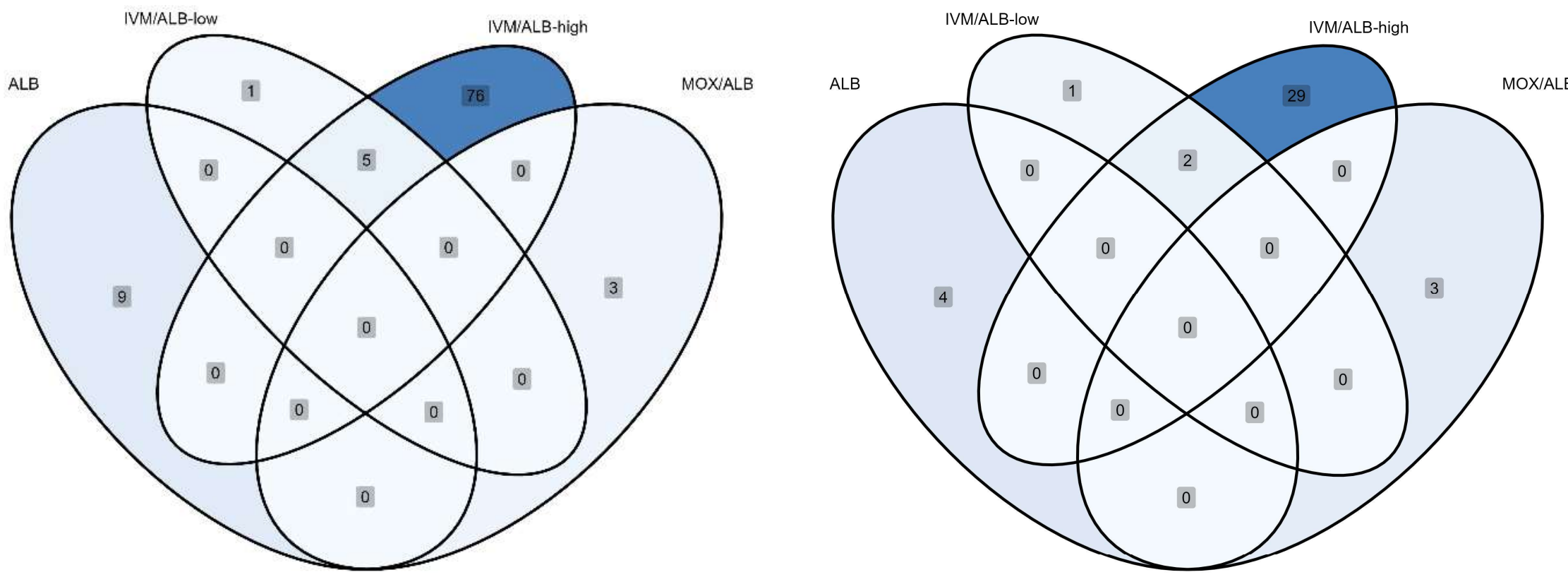

Supplementary Figure 21: Enriched or depleted pathways put forth by MaAsLin3 for each treatment arm. **Right:** Same enriched or depleted pathways after filtering, keeping only the associations with the 20% lowest and 20% highest MaAsLin3 coefficients and a relative signal > 0.5 for both abundance and prevalence modelling. The relative signal was calculated as the ratio of samples, in which the association was detected divided by the total number of samples for that arm and timepoint.

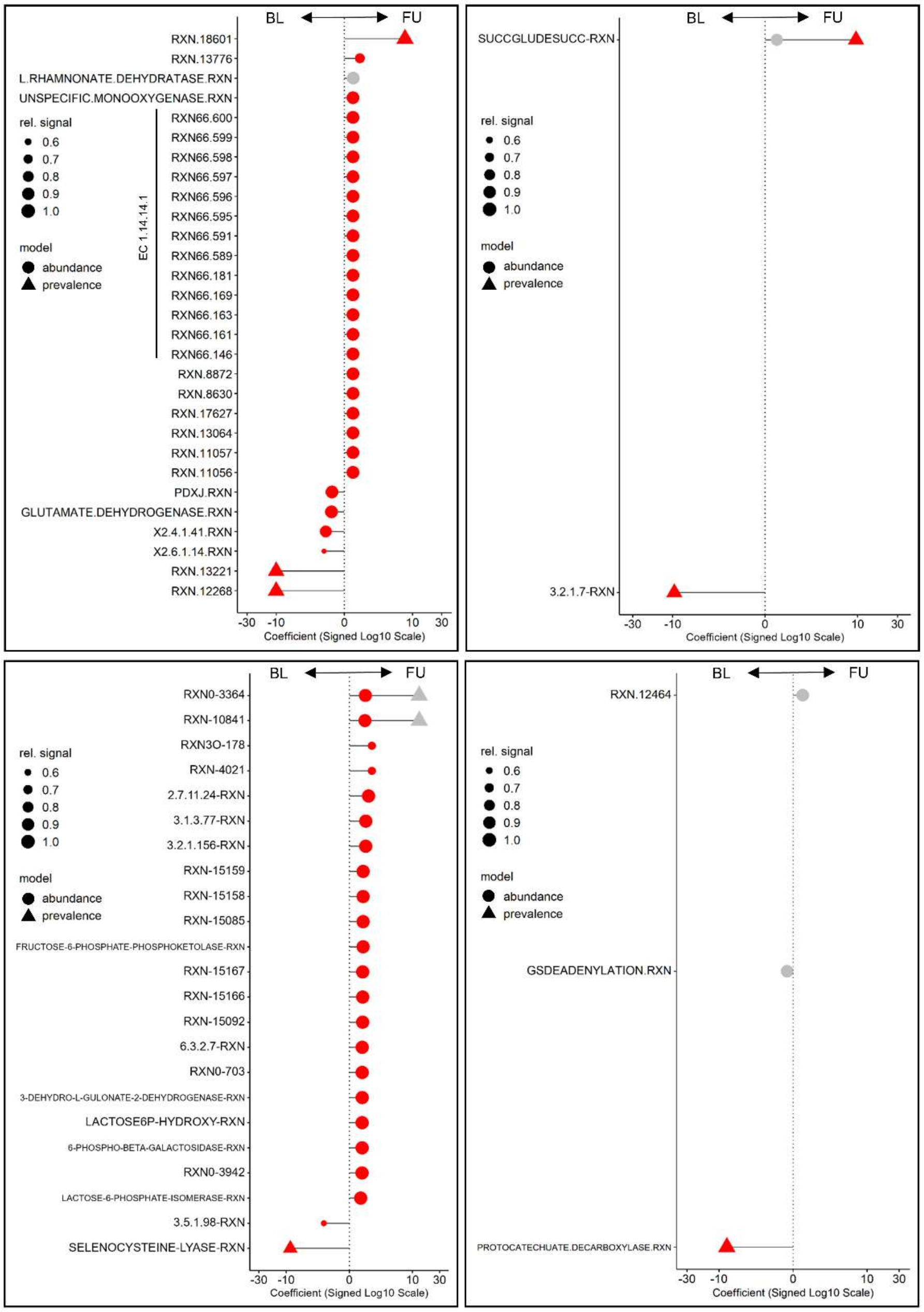

Supplementary Figure 22: Pairwise abundance and prevalence modelling of MetaCyc reactions between timepoints (BL = baseline, FU = follow-up) after filtering, keeping only the associations with the 5% lowest and 5% highest MaAsLin3 coefficients for both abundance and prevalence modelling and a relative signal > 0.5. Signals to the left of the x-axis centerline indicate association with BL, signals to the left of the x-axis centerline indicate association with FU. The relative signal was calculated as the ratio of samples, in which the association was detected divided by the total number of samples for that arm and timepoint. Only reactions reaching a "joint q-val" of < 0.1 are shown. Reactions with "individual q-val" < 0.1 are depicted in red. Coefficients for the abundance models are given as log2(FU/BL). Coefficients for the prevalence models are given as ln(odds-ratio(FU/BL)). **Top:** ALB (**left**), IVM/ALB-low (**right**). **Bottom:** IVM/ALB-high (**left**), MOX/ALB (**right**). Abbreviations: ALB, albendazole; IVM/ALB, ivermectin-albendazole; MOX/ALB, moxidectin-albendazole.

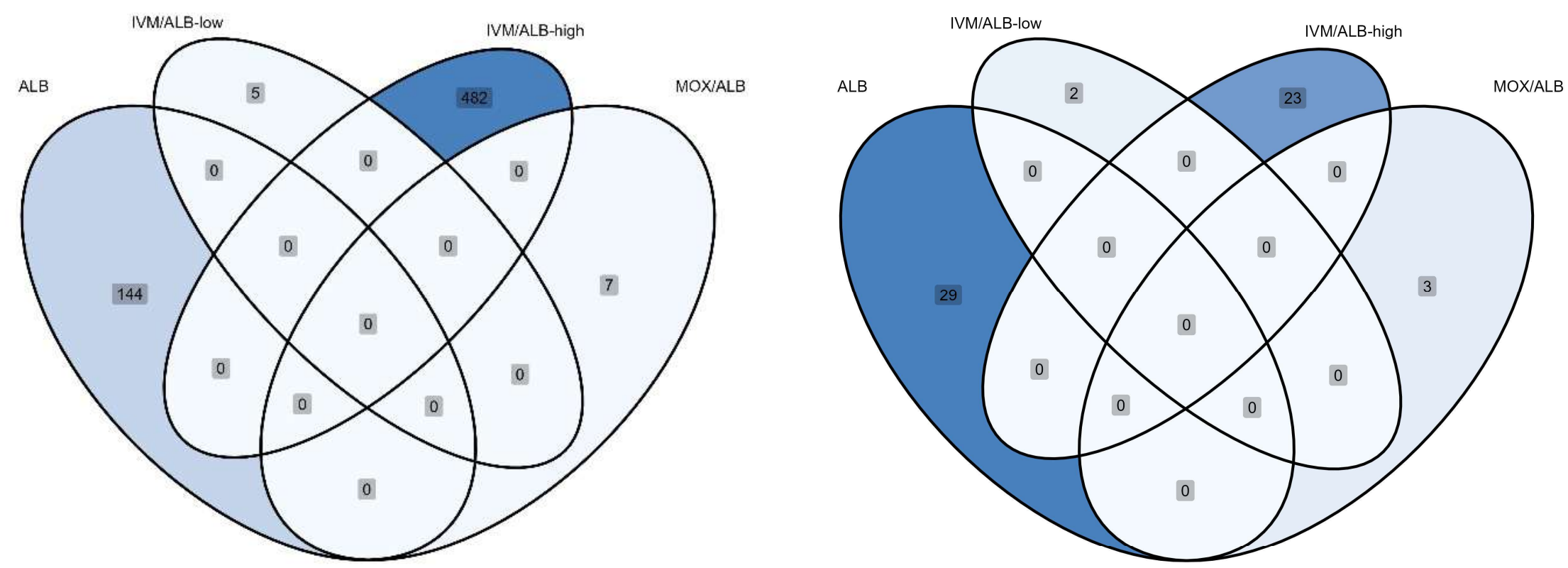

Supplementary Figure 23: Left: Enriched or depleted MetaCyc reactions put forth by MaAsLin3 for each treatment arm. **Right:** Same enriched or depleted reactions after filtering, keeping only the associations with the 5% lowest and 5% highest MaAsLin3 coefficients and a relative signal > 0.5 for both abundance and prevalence modelling. The relative signal was calculated as the ratio of samples, in which the association was detected divided by the total number of samples for that arm and timepoint.

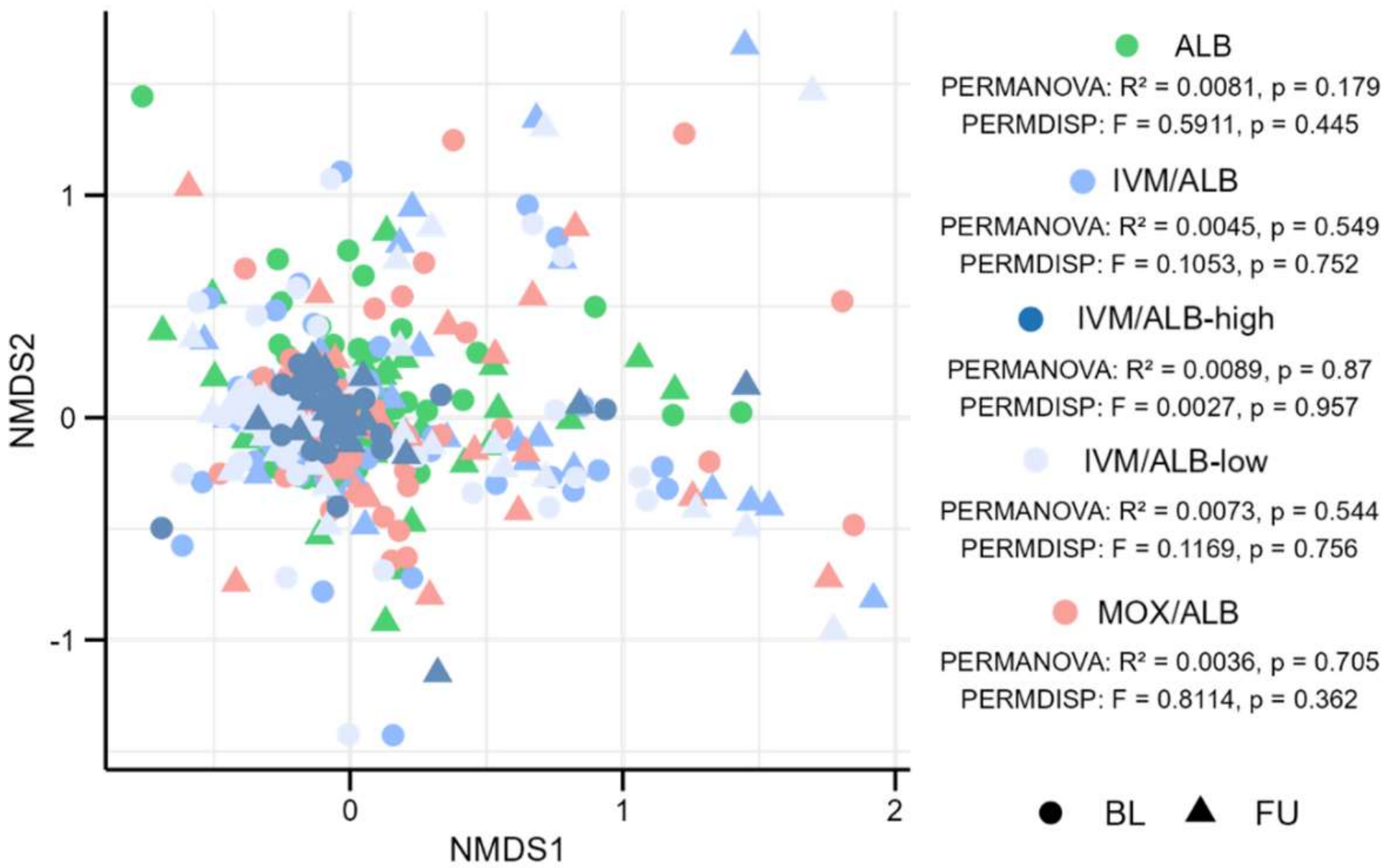

Supplementary Figure 24: Non-metric multidimensional scaling (NMDS) ordination based on Bray-Curtis Dissimilarity matrices of paired baseline (BL) and follow-up (FU) antimicrobial resistance gene abundances. The stratification of the IVM/ALB is based on the absolute administered dose of IVM (IVM/ALB-low: 3-12mg, IVM/ALB-high: 15-21mg). Abbreviations: ALB, albendazole; IVM/ALB, ivermectin-albendazole; MOX/ALB, moxidectin-albendazole.

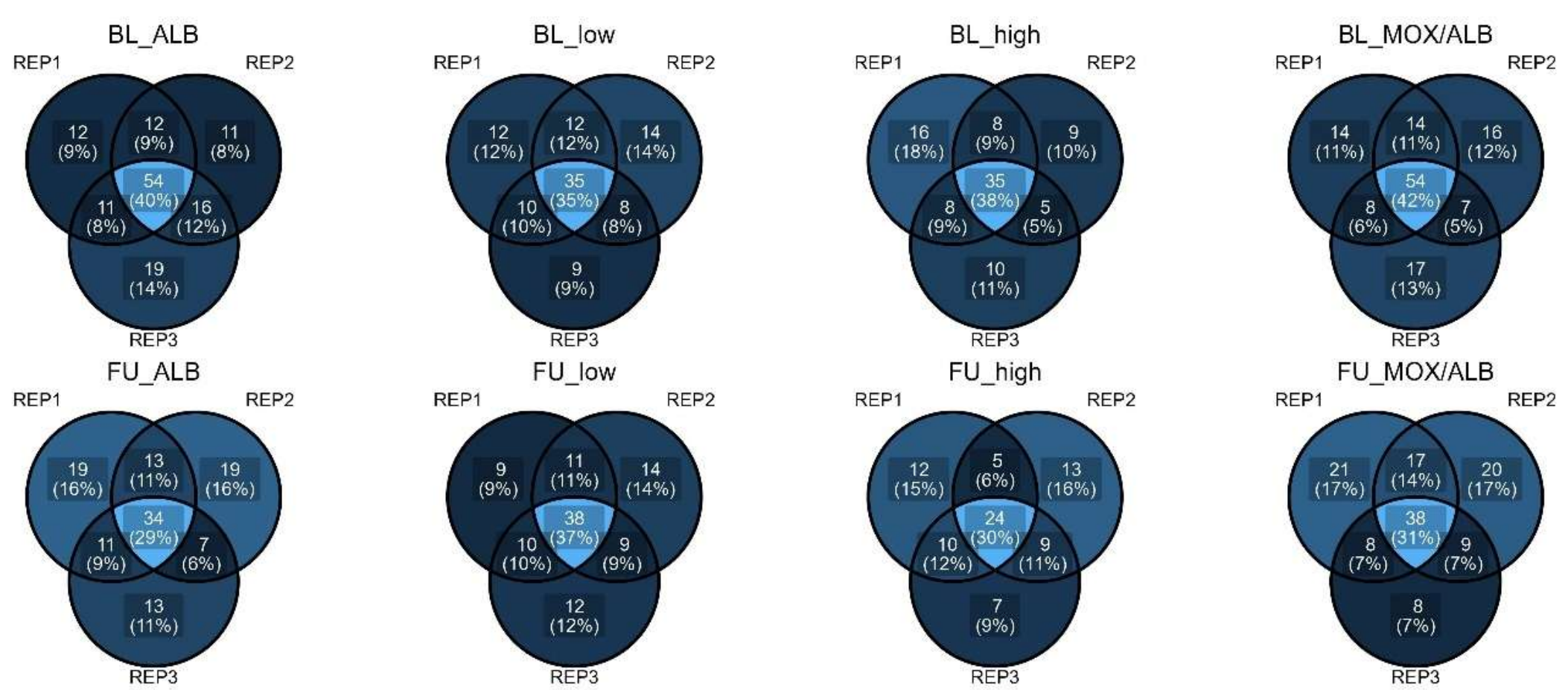

Supplementary Figure 25: Absolute number of shared and unique metagenomic bins for each replicate of a treatment arm (ALB, IVM/ALB-low, IVM/ALB-high, MOX/ALB) and timepoint (BL, FU). Abbreviations: BL, baseline; FU, follow-up; ALB, albendazole; IVM/ALB, ivermectin-albendazole; MOX/ALB, moxidectin-albendazole.
